# Mistletoe extract in patients with advanced pancreatic cancer: Health-related quality of life in a double-blind, randomized, placebo-controlled trial (MISTRAL)

**DOI:** 10.1177/02692163261437609

**Published:** 2026-04-30

**Authors:** Kathrin Wode, Ove Björ, Reinhild Klein, Nils Oskar Elander, Per Fransson, Roger Henriksson, Lena Sharp, Ursula Scheibling, Birgit Johansson, Johanna Hök Nordberg, Gunver Sophia Kienle

**Affiliations:** 1 Department of Diagnostics and Interventions/Oncology, Umeå University, Sweden; 2 Regional Cancer Centre Stockholm Gotland, Sweden; 3 Karolinska Institutet Department of Neurobiology, Care Sciences and Society, Stockholm, Sweden; 4 Department of Internal Medicine II, Immunopathological Laboratory, University Tübingen, Germany; 5 Department of Biomedical and Clinical Sciences and Department of Clinical Oncology, Linköping University, Sweden; 6 Department of Nursing, Umeå University, Sweden; 7 The Institute for Palliative Care, Lund University and Region Skåne, Sweden; 8 Department of Oncology, Ryhov County Hospital, Jönköping, Sweden; 9 Department of Oncology, Västmanlands Hospital, Västerås, Sweden; 10 Department of Internal Medicine II, Center for Complementary Medicine, Medical Center, Faculty of Medicine, University of Freiburg, Germany; 11 Institute for Applied Epistemology and Medical Methodology at Witten/Herdecke University (IFAEMM), Freiburg, Germany

**Keywords:** randomized controlled trial, quality of life, mistletoe, viscum album, complementary therapies, pancreatic neoplasms, standard of care, palliative medicine

## Abstract

**Background:**

Mistletoe extract is a widespread complementary therapy mainly used for quality-of-life improvement in cancer patients. Advanced pancreatic cancer is associated with poor quality of life and better therapies for symptomatic relief are highly needed.

**Aim:**

MISTRAL aimed to assess the impact of mistletoe extract on quality of life, body weight, observed costs and blood biomarkers in patients with advanced pancreatic cancer.

**Design:**

MISTRAL was an investigator-initiated, phase III, randomized, double-blind, placebo-controlled, parallel-group, superiority, multicenter, clinical trial with a nested biomarker study. Registration EudraCT 2014–004552–64, NCT02948309.

**Setting and participants:**

At 9 oncology centers, 290 participants were randomized to standard treatment (palliative chemotherapy or best supportive care) plus subcutaneous mistletoe extract or placebo. Main inclusion criteria were advanced pancreatic cancer, performance status 0–2, main exclusion criteria neuroendocrine pancreatic tumor. EORTC-QLQ-C30, EORTC-QLQ-PAN26, body weight, cost parameters and biomarkers were assessed from baseline up until 9 months.

**Results:**

No statistically significant differences for quality of life and weight were evident between treatment arms. Parameters for observed costs for supportive and inpatient care (days at hospital, parenteral nutrition infusions, nutritional supplement drinks, number of visits of palliative home care teams, symptom-relieving medication) were similar in both arms. Thus, calculation of costs was not performed. No effect on explored biomarkers (differential blood count, lymphocyte subpopulations, C-reactive protein, albumin and Ca19-9) was found except for a statistically significant increase of eosinophils in the mistletoe arm without association to clinical effect.

**Conclusions:**

Since no benefit was observed, there is no clinical reason to recommend mistletoe extract in patients with advanced pancreatic cancer.

**What is already known about the topic?**

- Mistletoe extract is one of the most common complementary cancer therapies used in addition to standard care in both curative and palliative situations.
- Previous studies have suggested a moderate impact on quality of life, increased blood levels of immunocompetent cells, reduced need for symptom relieving drugs, less hospitalizations and lower costs for hospital stays in various types of cancer.
- Some studies have suggested a substantial impact of subcutaneous mistletoe extract on quality of life and gain of body weight in patients with advanced pancreatic cancer.

**What this paper adds**

- No significant impact on health-related quality of life (as per EORTC-QLQ-C30 and EORTC-QLQ-PAN26 assessment) or body weight was found.
- Parameters for observed costs for supportive and inpatient care were comparable in both treatment arms. Costs were thus not further calculated.
- No effect on biomarkers (differential blood count including lymphocyte subpopulations and serum levels of C-reactive protein, albumin, and Ca19-9) was found except for a significant increase of eosinophils in the mistletoe arm. However, this had no association with clinical outcome.

**Implications for practice**

- Since no benefit was observed, there is no clinical reason to recommend mistletoe extract in patients with advanced pancreatic cancer.

## Background

Advanced pancreatic cancer is associated with poor quality of life^1,2^ and, together with improved tumor specific treatments, optimized symptom management is essential in a comprehensive cancer care context.^3^

Mistletoe extract is widely used as a complement to cancer-specific and evidence-based treatment, particularly in Europe. Increasing use is reported (but not systematically quantified) from other countries such as South America, USA and Canada.^4–6^ Raised blood levels of leukocytes, granulocytes, natural killer cells, eosinophils and lymphocyte subsets have been shown and proposed to be a mechanism of action of subcutaneous mistletoe extract application.^7–12^ However, it is still unclear whether these changes correlate with a clinical response to mistletoe extract. Prior studies demonstrated a moderate impact on quality of life in various types of cancer.^13,14^ A reduced need for symptom reliving drugs,^15^ less frequency of hospitalizations^16^ and lower costs for hospital stays^17^ have also been shown. Regarding advanced pancreatic cancer, an open-label randomized controlled trial (MAPAC) reported a substantial impact of subcutaneous mistletoe extract on overall survival, quality of life and gain of body weight.^18,19^

Following these promising results of the MAPAC study, the randomized phase III multicenter double-blind MISTRAL trial was designed and conducted to assess mistletoe extract as a complement to standard treatment in advanced pancreatic cancer.^20^ No effect of mistletoe extract compared to placebo was found in terms of the primary endpoint overall survival.^21^ Here, we present findings for secondary endpoints including health-related quality of life (further on quality of life), body weight, and parameters for costs for supportive and inpatient care as well as blood biomarkers (differential blood count including lymphocyte subpopulations and serum levels of C-reactive protein, albumin, and Ca19-9).

## Methods

### Research question/hypothesis

The aim of this study was to examine whether mistletoe extract as a complement to standard treatment (palliative chemotherapy or best supportive care) might improve quality of life and body weight and reduce the observed costs for supportive and inpatient care. Immunological effects and potential prognostic and predictive blood biomarkers were explored.

### Design

MISTRAL was an investigator-initiated, phase III, randomized, double-blind, placebo-controlled, parallel group, superiority, multicenter, clinical trial with a nested ancillary biomarker study on sub-sets of participants. For details see study protocol.^20^

### Setting

This study was conducted at nine Swedish oncology centers; at three centers, a sub-set of the trial’s cohort was included in the nested ancillary biomarker study.

### Population

Main inclusion criteria were recent diagnosis of advanced pancreatic cancer or relapse, Eastern Cooperative Oncology Group performance status ⩽2 and life expectancy >4 weeks. Main exclusion criterion was neuroendocrine tumor of the pancreas. All participants received standard of care including symptom management with or without palliative chemotherapy. A sample size of 290 was required to achieve 90% power at a 5% significance level in a two-sided log rank test to detect a HR of 0.67 between treatment arms for the primary endpoint survival.

### Randomization

Participants were randomly assigned to receive mistletoe extract or placebo in a 1:1 ratio, stratified by site and palliative chemotherapy (eligible or not).

### Recruitment

Potential participants were identified at multidisciplinary tumor conferences or at therapy conferences at participating centers and at associated palliative home care units.

### Intervention

The study drug (fermented aqueous extract of Viscum album L. grown on oak tree) or placebo (isotonic saline solution) was injected subcutaneously thrice weekly for 9 months. Dosage was stepwise increased and individually adapted from 0.01 over 0.1, 1 and 10 mg to a maximum of 20 mg. Dose escalation and adaptation of maintenance dose followed established clinical use, treatment guidelines of the manufacturer and the MAPAC trial using dose escalation from 0,01 over 0.1, 1 and 5 mg to a maximum of 10 mg.^19,20,22,23^

### Data collection

Follow-up was performed at week 5–6 and subsequently at 2, 3, 4, 6 and 9 months after randomization. At each of the seven study visits, quality of life using the EORTC-QLQ-C30^24^ and EORTC-QLQ-PAN-26^25^ questionnaires, body weight, concomitant medication, and number of days at hospital since last visit were documented.

Information from study-specific diaries was recorded, including participants’ weekly documentation of body weight at home, the number of administered total parenteral nutrition infusions, the average consumption of dietitian-prescribed nutritional supplement drinks, and the number of visits by their palliative home care team.

Within the biomarker study, blood samples for analysis of differential blood count, lymphocyte subpopulations, C-reactive protein, albumin and Ca19-9 were collected before first injection with study drug at baseline visit and after month 1, 2, 3 to 4 and 9 to 10.

### Data analysis

Details of statistical analyses are specified in the Statistical Analysis Plan (approved 22 December 2022). Change from baseline to follow-up visits of each quality-of-life dimension was compared between study arms. Analysis included participants who completed the quality-of-life questionnaire at trial start and at least one follow-up visit. Absolute changes in quality-of-life scores from baseline to follow-up visits were analyzed and compared. Linear mixed-effects models with random intercepts for each patient were included, allowing inclusion of all available observations under a missing-at-random assumption without imputation. The models included “treatment” (mistletoe extract or placebo) and “study visit” as fixed factors, both treated as categorical variables, and were adjusted for baseline quality-of-life scores. We also examined treatment and study visit interactions using mixed models.

For analysis of relative weight changes a corresponding mixed model approach as for quality of life was used. Only weights recorded within 14 days of the expected visits at week five-six, month two, three, four, six and nine were included. Body weight measurement recorded in patient diaries took preference over weights measured at study visits. If diary weights were unavailable or differed by more than 14 days from the expected date, body weight recorded at the corresponding study visit was used —given the visit occurred within 14 days of the expected date.

Costs for supportive care and inpatient care were planned to be calculated based on the following parameters: concomitant medication, days in hospital, visits from palliative home care team and administration of parenteral nutrition and consumption nutritional supplement drinks.

Biomarkers including differential blood count, C-reactive protein, albumin, and Ca19-9 were analyzed by standard methods. Lymphocyte subpopulations were measured by FACS -analysis (CD3+ T cells, CD19+ B cells, CD4+/CD3+ helper T cells, CD8+/CD3+ cytotoxic T cells, CD19+ B-cells, CD16+/CD56+ NK cells). The non-parametric Mann-Whitney test was used for unpaired data (comparison of mistletoe extract and placebo), and the non-parametric Wilcoxon-test for paired data (comparison baseline before start with injections and measurements after month 1, 2, 3 to 5 and 9 to 10). Patient safety data (adverse events and severe adverse events) have previously been analyzed and presented per treatment arm in.^21^

### Ethics and patient consent

Stockholm’s Regional Ethical Review Board approved the trial (2 March 2016, 2016/122–31/2) and amendment nr 1 (24 November 2016, 2016/2272-32) for the ancillary biomarker study. The Declaration of Helsinki and Good Clinical Practice were implemented. Written informed consent was obtained from all participants before any trial-related procedures took place. Trial registration: EudraCT 2014–004552–64, NCT02948309.

## Results

**Figure 1.**
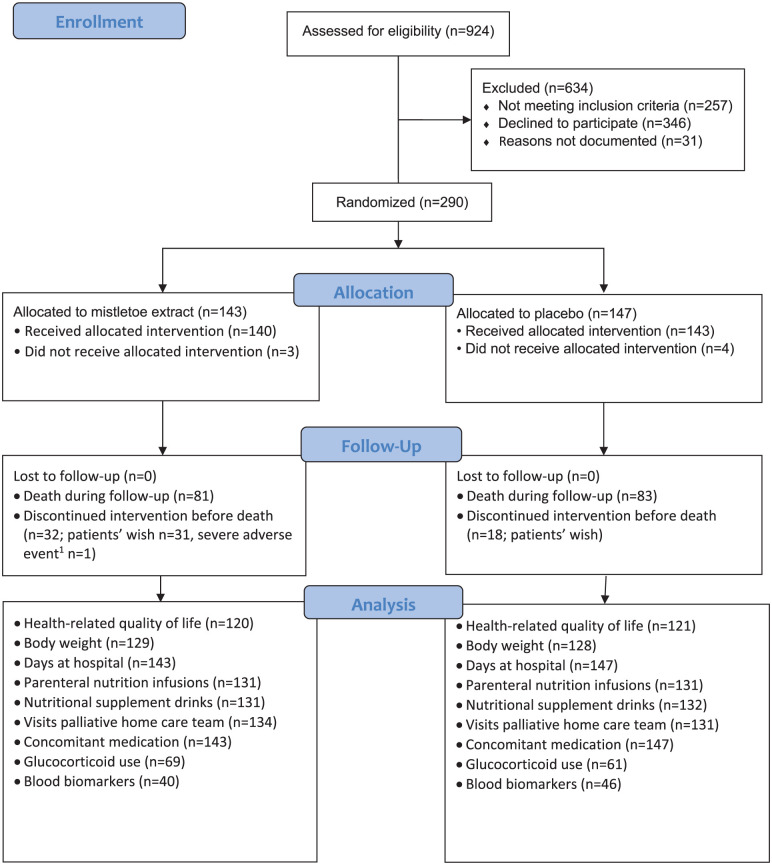
Flowchart of trial participants. ^1^Urticaria (known side-effect from mistletoe extract).

**Figure 2.**
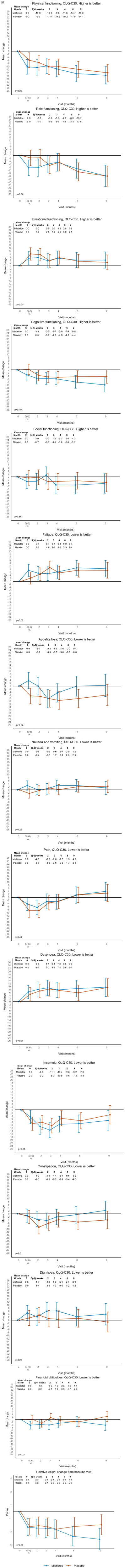
Mean changes in quality-of-life and weight change by treatment arm and visit. Changes in quality of life scores for five of six^1^ functional scales and nine symptom scales compared to baseline scores (95% confidence interval), calculated using mixed model regression. Data was collected using the EORTC-QLOQ-C30 questionnaire. Blue mistletoe extract, red placebo. Higher scores indicate better quality of life for physical, role, emotional, cognitive and social functioning Lower scale indicate better quality of life for fatigue, appetite loss, nausea and vomiting, pain, dyspnea, insomnia, constipation, diarrhea and financial difficulties. *p*-value from test of the treatment effect by Satterthwaite’s method is shown in the figure. Marginal mean weight change (95% CI) in percent from weight registered at baseline visit estimated from mixed model. ^1^Results for the functional scale global health have been published in (Wode, 2024 #5018).

From 7 June 2016 to 3 December 2021, 290 patients were included and randomized to two study arms (Figure 1). Baseline characteristics were well balanced except for slight differences in distributions of tumor stages and primary diagnosis versus relapse (Table 1). Quality of life was assessed in 241 participants (*n* = 120 mistletoe and *n* = 121 placebo arm). Details on visits and questionnaire coverage have been published in.^21^ Overall, quality of life varied over time and with progressing disease most symptoms deteriorated with negligible differences between study arms. The clinical relevance of the deterioration observed could, overall, be described as small to moderate.^26^ No advantage for the mistletoe arm was seen in any of the quality-of-life dimensions from the EORTC-QLQ-C30 and EORTC-PAN-26 questionnaires; the only statistically significant difference between arms was slightly more loss of appetite in the mistletoe arm (Figure 2, Supplemental Table 1, Supplemental Figure 1).

**Table 1.** Baseline characteristics for participants.

Patient characteristics	ME	Placebo
Patients, *n*	120	121
Sex, female, *n*	59	61
Age, mean (SD)	69.0 (9.1)	67.2 (10.4)
Primary diagnosis advanced pancreatic cancer, *n*	93	80
Relapse of pancreatic cancer, *n*	27	41
Clinical/pathological T stage, *n*		
T0/Tis	0	0
T1	3	1
T2	7	12
T3	27	22
T4	49	38
TX	7	7
EORTC QLQ-30, mean (SD)^d^		
Physical functioning	78.6 (17.6)	77.5 (20.9)
Role functioning	65.7 (30.0)	62.9 (34.1)
Emotional functioning	73.2 (21.2)	73.7 (20.4)
Cognitive functioning	84.9 (18.7)	84.7 (19.0)
Social functioning	70.3 (26.0)	67.6 (31.0)
Fatigue	40.3 (24.0)	40.2 (26.3)
Appetite loss	39.0 (35.2)	33.6 (34.6)
Nausea and vomiting	11.9 (16.1)	12.1 (19.7)
Pain	28.9 (26.4)	30.9 (29.1)
Dyspnea	23.2 (24.4)	23.7 (28.4)
Insomnia	26.7 (26.8)	30.6 (28.7)
Constipation	25.8 (31.0)	20.0 (29.1)
Diarrhea	22.7 (30.4)	21.4 (29.9)
Financial difficulties	8.1 (20.8)	12.2 (24.4)
EORTC PAN-26, mean (SD)		
Pancreatic pain	29.4 (22.8)	27.6 (21.5)
Digestive symptoms	34.6 (31.8)	29.5 (28.8)
Hepatic	5.6 (13.7)	7.6 (14.2)
Altered bowel habit	23.5 (25.9)	23.9 (26.3)
Body image	27.9 (30.3)	26.0 (28.6)
Satisfaction with health care	17.4 (21.1)	23.1 (26.2)
Sexuality	60.1 (36.9)	56.2 (37.5)
Did you have a bloated feeling in your abdomen?	34.2 (31.6)	28.5 (30.3)
Did food and drink taste different from usual?	33.3 (36.4)	29.4 (33.2)
Have you had indigestion?	33.3 (31.1)	23.6 (29.7)
Were you bothered by gas (flatulence)?	37.2 (31.5)	41.0 (33.4)
Have you worried about your weight being too low?	30.6 (33.1)	29.6 (31.2)
Did you feel weak in your arms and legs?	31.9 (28.8)	26.8 (29.3)
Did you have a dry mouth?	41.7 (35.0)	35.9 (30.9)
To what extent have you been troubled with side-effects?	22.9 (26.9)	25.1 (31.0)
Were you worried about your health in the future?	58.0 (29.3)	61.3 (31.3)
Were you limited in planning activities, for example meeting friends?	42.9 (35.7)	43.0 (34.5)
ECOG performance status^b^, *n* (%)		
0	53 (44.2%)	61 (50.4%)
1	52 (43.3%)	41 (33.9%)
2	15 (12.5%)	19 (15.7%)
Body weight at baseline visit, mean (SD)	70.5 (12.5)	71.8 (16.9)
Connected to palliative home care team^c^, *n* (%)	54 (45%)	35 (29%)

Data presented as *n* (SD). Characteristics assessed for health-related quality of life (*n* = 241).^a^

a Based on participants included in the follow-up analysis data set with measurement of Health-related quality of life at baseline and for at least one follow-up.

b From baseline visit at first hand, else from randomization.

c At baseline visit.

In total 257 participants had their baseline weight and at least one follow-up weight registered within 14 days from the expected study visit (*n* = 129 mistletoe and *n* = 128 placebo arm). For relative change in body weight (follow-up vs baseline), no statistically significant difference between study arms was evident. Overall decrease in weight estimated from mixed model regression was –2.8 kg (−4.0; −1.6) for mistletoe extract and –2.3 kg (−3.5; −1.2) for placebo. Overall difference in weight-change between mistletoe arm and placebo arm was −0.5 kg (−1.7; 0.7; Figure 2, Supplemental Table 2).

Average number of days spent in hospital was assessed for all 290 participants. Results were similar both in terms of average number of days (8 in the mistletoe arm; 7.7 in the placebo arm), percentage of days in the study (0.9% in mistletoe arm; 0.8% in the placebo arm) and average number of days since previous visit (Supplemental Tables 3–4). Average number of consumed dietitian-prescribed nutritional supplement drinks per patient was similar in absolute numbers during the entire study period (15.7 mistletoe and 13.6 placebo arm) and as average number of drinks per month in study (2.7 mistletoe arm; 2.1 placebo arm; Table 2). Comparable results were found for the average number of infusions with total parenteral nutrition in absolute numbers during the entire period of study participation (6.1 mistletoe and 5.0 placebo arm) and in relation to months in study (1.1 mistletoe and 0.8 placebo arm; Table 3). Furthermore, number of visits from palliative home care teams during study participation was similar between study arms both in absolute numbers (23.7 mistletoe and 25.1 placebo arm) and in relation to months in study (4.2 mistletoe and 3.9 placebo arm; Table 4).

**Table 2.** Dietician-prescribed nutritional supplement drinks.

Teatment	Total number of months in study	Total number of nutritional drinks	Number of patients	Average number of nutritional drinks per patient	Average number of nutritional drinks per months in study^a^
Mistletoe extract	756	2057	131	15.7	2.7
Placebo	849	1797	132	13.6	2.1
Total	1605	3853	263	14.7	2.4

Average number of consumed drinks in absolute numbers and in relation to time (months) in study. Data derived from study-specific patient diaries with weekly documentation of average number consumed nutritional supplement drinks per day. In 27 diaries, information on nutritional supplement drinks is missing.

a Time in study is defined as time from baseline visit to discontinued intervention or end of 9-months treatment period.

**Table 3.** Infusions with total parenteral nutrition.

Treatment	Total number of months in study	Total number of infusions given	Number of patients	Average number of infusions given per patient	Average number of infusions given per month in study^a^
Mistletoe extract	744	804	131	6.1	1.1
Placebo	842	653	131	5.0	0.8
Total	1586	1457	262	5.6	0.9

Average number of infusions in absolute numbers and in relation to time (months) in study. Data derived from study specific patient diaries with weekly documentation of number administered nutritional during the preceding week. In 28 diaries, information on total parenteral nutrition is missing.

a Time in study is defined as time from baseline visit to discontinued intervention or end of 9-months treatment period.

**Table 4.** Visits from palliative home care teams.

Treatment	Total number of months in study	Total number of home visits	Number of patients	Average number of home visits per patient	Average number of home visits per months in study
Mistletoe extract	751	3172	134	23.7	4.2
Placebo	848	3287	131	25.1	3.9
Total	1599	6459	265	24.4	4.0

Average number of home visits in absolute numbers and in relation to time (months) in study. Data derived from study specific patient diaries with weekly documentation of number of visits from palliative home care teams independent of profession (e.g. nurse, doctor, physiotherapist, counselor) during the preceding week. In 25 diaries, information on visits by palliative home care teams was missing.

Concerning the use of concomitant medication for symptom relief and patient-initiated use of complementary natural products, vitamins and minerals, no obvious difference between study arms was revealed (Supplemental Tables 5 and 6).

Treatment patterns of glucocorticoids for symptom management were analyzed. The number of participants using glucocorticoids was comparable in both arms (*n* = 69 mistletoe, *n* = 61 placebo), as well as median use in months (1.15 mistletoe and 1.12 placebo arm; Supplemental Table 7) and percent of time in study (Supplemental Figure 2).

From April 2018 to December 2021, a subset of 86 participants from the MISTRAL population (*n* = 40 mistletoe and *n* = 46 placebo arm) was included in the ancillary biomarker study. A significant difference (*p* < 0.05) between study arms was evident in terms of higher eosinophil counts month 2–10 in the mistletoe arm particularly in participants receiving chemotherapy (unpaired analysis), month 3–5 (mistletoe arm) and month 9–10 (placebo arm) in paired analysis (Supplemental Figure 3(a), Supplemental Tables 8–10). To assess a potential difference in immunostimulation in relation to standard treatment, participants treated with chemotherapy (*n* = 28 mistletoe and *n* = 31 placebo arm) and those treated with best supportive care (*n* = 12 mistletoe and *n* = 15 placebo arm) were analyzed separately within study arms: participants receiving chemotherapy had significantly higher levels of eosinophils, lymphocytes and basophils than those with best supportive care (Supplemental Figure 4(a) and (b), Supplemental Table 10). Tumor marker Ca-19-9, C-reactive protein and albumin were not influenced by mistletoe extract. (Supplemental Figures 3(b) and 4(c) and (d), Supplemental Tables 9 and 11).

Regarding safety data (for details see^21^), event rates by time in study and severity of adverse events and serious adverse events were comparable in both treatment arms (46% of patients in the mistletoe and 45% in the placebo arm reported at least one adverse or serious adverse event). Two severe adverse events (one urticaria, one pseudo allergic reaction) were both assessed as related to the study drug (one of them leading to discontinuation of the study, Figure 1).

## Discussion

### Main findings/results of the study

No significant differences in terms of quality of life and body weight were evident when comparing mistletoe extract and placebo treatment as addition to standard of care (palliative chemotherapy or best supportive care) in patients with advanced pancreatic cancer. Furthermore, no impact of mistletoe extract was found on the use of glucocorticoids for symptom management, other concomitant medication, days at hospital, visits of palliative home care teams, administered total parenteral nutrition infusions or consumption of dietitian-prescribed nutritional supplement drinks. Thus, it was not applicable or relevant to further analyze potential cost differences in terms of supportive and inpatient care in the respective arms. No impact on biomarkers (differential blood count, lymphocyte subpopulations, C-reactive protein, albumin and Ca19-9) except for increased levels of blood eosinophils in the mistletoe arm was found.

### What this study adds

As discussed earlier,^21^ prior research on mistletoe extract in cancer suggested an improvement of quality of life^13,14,18^ and survival^19,27^ as well as a reduction of cancer care-related costs.^16,17^ Improved quality of life was reported in two placebo-controlled trials on breast cancer,^28,29^ and the MAPAC trial on advanced pancreatic cancer suggested a significant impact on quality of life and body weight; the latter study was, however, performed without blinding patients or study personnel and was conducted in a Serbian healthcare context that might differ from a Scandinavian setting of comprehensive cancer care including full access to home-based palliative care.^18,19^ We intended to explore the potential impact of mistletoe extract in a modern and more rigorous trial in a country with high quality cancer care. Our results do not confirm the quality-of-life results from the MAPAC study although, notably, the same questionnaire (EORTC-QLQ-C30) was used in both trials. The slightly worse outcome observed for appetite loss in the mistletoe arm of the MISTRAL trial (*p* = 0.02) may potentially be caused by small differences in the distribution of more advanced tumor stages (T4) in participants with a primary diagnosis of pancreatic cancer.

To assess whether a potential impact of mistletoe extract was masked by unbalanced or biased symptom management between study arms, concomitant medication was analyzed. Glucocorticoids are commonly used in late-stage pancreatic cancer for appetite stimulation, pain relief and for general improvement of subjective physical and mental energy levels. In addition, chemotherapy itself may be associated with improved quality of life depending on tumor response and tolerability. As exposure to glucocorticoids, other symptom-relieving drugs and chemotherapy (the latter results presented in^21^) was similar in both study arms, these findings support that there was no relevant effect of mistletoe extract on quality of life in the present study.

Increased levels and/or activity of immune competent cells in response to mistletoe extract has been proposed to be a beneficial and plausible mode of action in terms of symptomatic control.^7–12^ We therefore analyzed blood samples to explore a possible immune response. The only significant difference evident was increased blood levels of eosinophils amongst patients in the mistletoe arm. This finding was independent of treatment with chemotherapy or best supportive care. No effect of mistletoe extract on any other of the analyzed blood cell types was found. Neither were any differences with regards to CRP, serum albumin or CA19-9 levels in the respective arm observed. These results might possibly be explained by the low immunogenicity and strong immunosuppressive mechanisms of pancreatic tumors.^30–34^

MISTRAL was the first blinded trial on mistletoe extract and quality of life in pancreatic cancer. Even though blinding is considered important in randomized controlled trials minimizing the potential influence of participants’ and clinicians’ expectations, accumulating evidence suggests that blinding per se may not be mandatory for patient-reported outcomes.^35–37^ This is in line with our findings of local skin reactions in MISTRAL: although 66% of patients in the mistletoe arm and just 1% of patients in the placebo arm experienced any local reaction (that in theory might have affected patients’ and clinicians belief on randomization to mistletoe extract or placebo), this did however not seem to have affected how participants precepted or reported their quality of life in this trial.

### Strengths and weaknesses/limitations of the study

Strengths of the present study include the placebo-controlled design, the published study protocol conforming to SPIRIT guidelines,^20^ the multicenter design in an academic setting, public and non-profit funding, trial conduct in a country with comprehensive oncological and palliative care and a multidisciplinary research team of experts specializing in oncology, pharmacology, nursing, integrative medicine, and palliative care. Robustness of the dataset was assured by quality controls, adherence to the treatment schedule and high return of questionnaires and study-specific diaries (^21^ for details). All data are comprehensively analyzed and presented. The present study results are consistent with the earlier reported primary outcomes of no apparent effect of mistletoe extract on overall survival compared to placebo (AHR 1.13; 1.44).^21^ Although the study was primarily powered to assess overall survival, the between-arm differences in deterioration were generally modest, rarely exceeding five units.^26^ Treatment settings enabling patients’ active involvement in their therapy have been assumed to be beneficial beyond the specific treatment effect.^38^ External validity for quality of life may thus be reduced due to the randomized design since mistletoe treatment was stripped from a context where patients usually actively seek out this therapy.^39,40^

## Conclusion

Up to now, a benefit of mistletoe extract on quality of life among patients with advanced pancreatic cancer was considered to be possible, with good tolerability of the treatment. As with the presented study, which results are unbiased, based on rigorous methodology and a thorough dataset with ensured data quality, benefit from mistletoe extract on quality of life, body weight and observed costs for supportive and inpatient care could not be confirmed. Logically, no blood biomarker was identified predicting any effect of mistletoe extract. In palliative care, disease-related symptoms are not regarded to be tumor- specific and are generally treated independently of the tumor entity. Consequently, not observing any effect on quality of life in this trial raises questions about whether mistletoe extract can substantially improve quality of life in other cancer types, as is commonly assumed from prior results from clinical trials in other tumors. These studies are older and from other treatment contexts and should therefore be re-investigated in randomized controlled trials embedded in the context of modern cancer care.

Together with our previously published negative results on overall survival, we conclude that there is no clinical reason to recommend mistletoe extract in patients with advanced pancreatic cancer.

## Supplemental Material

sj-docx-1-pmj-10.1177_02692163261437609 – Supplemental material for Mistletoe extract in patients with advanced pancreatic cancer: Health-related quality of life in a double-blind, randomized, placebo-controlled trial (MISTRAL) Supplemental material, sj-docx-1-pmj-10.1177_02692163261437609 for Mistletoe extract in patients with advanced pancreatic cancer: Health-related quality of life in a double-blind, randomized, placebo-controlled trial (MISTRAL) by Kathrin Wode, Ove Björ, Reinhild Klein, Nils Oskar Elander, Per Fransson, Roger Henriksson, Lena Sharp, Ursula Scheibling, Birgit Johansson, Johanna Hök Nordberg and Gunver Sophia Kienle in Palliative Medicine
