# Supplementary material for: Mistletoe extract in patients with advanced pancreatic cancer: Health-related quality of life in a double-blind, randomized, placebo-controlled trial (MISTRAL)
Source: Palliat Med. 2026 Apr 30;40(7):1059–76. doi: 10.1177/02692163261437609 (PMC13323928; doi:10.1177/02692163261437609)
Supplement: sj-docx-1-pmj-10.1177_02692163261437609 – Supplemental material for Mistletoe extract in patients with advanced pancreatic cancer: Health-related quality of life in a double-blind, randomized, placebo-controlled trial (MISTRAL) [file sj-docx-1-pmj-10.1177_02692163261437609.docx]

# Supplement

## Mistletoe Extract in Patients with Advanced Pancreatic Cancer: Health-Related Quality of Life in a Double-blind, Randomized, Placebo-Controlled Trial (MISTRAL)

K. Wode, O. Björ, R. Klein, N. O. Elander, B. Johansson, U. Scheibling, L. Sharp, R. Henriksson, P. Fransson, J. Hök Nordberg, G. S. Kienle

Table of Contents

[S-Table 1: Mean change from baseline by visit and treatment effects on health-related quality of life -dimensions 2](#_Toc220587508)

[S-Figure 1: Mean changes in quality of life scores for symtom scales. 4](#_Toc220587509)

[S-Table 2: Test of difference 13](#_Toc220587510)

[S-Table 3: Days in hospital during the study 13](#_Toc220587511)

[S-Table 4: Days in hospital since previous visit 14](#_Toc220587512)

[S-Table 5: Concomitant medication for symptom relief 14](#_Toc220587513)

[S-Table 6: Patient-initiated use of natural products, vitamins and minerals^1^ 15](#_Toc220587514)

[S-Table 7: Glucocorticoid use for symptom management^1^. 15](#_Toc220587515)

[S-Figure 2: Glucocorticoid use by time in study per indication 16](#_Toc220587516)

[S-Figure 3 A-B: Eosinophil counts and albumin levels 17](#_Toc220587517)

[S-Figure 4 A-D: Eosinophil- and lymphocyte counts, Ca19-9 and albumin levels 18](#_Toc220587518)

[S-Table 8: Comparison of laboratory parameters in mistletoe extract versus placebo arm, unpaired analysis 20](#_Toc220587519)

[S-Table 9: Comparison of laboratory parameters in mistletoe extract versus placebo arm, paired analysis. 21](#_Toc220587520)

[S-Table 10: Comparison of laboratory parameters in relation to treatment with best supportive care versus chemotherapy regardless treatment arm, unpaired analysis. 22](#_Toc220587521)

[S-Table 11: Comparison of p-values for laboratory parameters for best supportive care versus chemotherapy in mistletoe extract and placebo arm, paired analysis 23](#_Toc220587522)

## S-Table 1: Mean change from baseline by visit and treatment effects on health-related quality-of-life dimensions

For coverage of follow-up analysis set by visit see [1]. The number of Participants with follow-up data decreased over time as many participants (n=81 mistletoe extract arm; n= 83 placebo arm) died during study participation.

|  | Overall Mean change from baseline by Visit ^a^ | |  | Number of patients ^c^ | |
| --- | --- | --- | --- | --- | --- |
| Item | mistletoe extract (95% CI) | Placebo (95% CI) | Treatment effect (95% CI) ^b^ | mistletoe extract | Placebo |
| QLQ-C30 Functional scales ^d^ |  |  |  |  |  |
| Physical functioning | -12.3 [-16.8; -7.9] | -9.8 [-14.2; -5.4] | -2.5 [-6.5; 1.5] | 120 | 121 |
| Role functioning | -8.9 [-13.3; -4.6] | -6.1 [-10.3; -1.9] | -2.8 [-8.8; 3.2] | 120 | 121 |
| Emotional functioning | 4.1 [0.0; 8.1] | 5.2 [1.5; 8.8] | -1.1 [-4.6; 2.5] | 120 | 120 |
| Cognitive functioning | -6.3 [-9.1; -3.5] | -3.7 [-6.4; -1.0] | -2.6 [-6.4; 1.3] | 120 | 120 |
| Social functioning | -3.4 [-7.3; 0.5] | -2.2 [-6.0; 1.6] | -1.2 [-6.7; 4.2] | 120 | 119 |
| QLQ-C30 Symptom scales ^e^ |  |  |  |  |  |
| Fatigue | 7.3 [4.0; 10.6] | 6.0 [2.8; 9.2] | 1.3 [-3.2; 5.9] | 120 | 121 |
| Appetite loss | -1.2 [ -5.4; 2.9] | -7.8 [-11.7; -3.8] | **6.5 [0.9; 12.2]^f^** | 118 | 121 |
| Nausea and vomiting | 2.2 [-0.2; 4.6] | 0.2 [-2.1; 2.6] | 2.0 [-1.4; 5.3] | 120 | 121 |
| Pain | -1.5 [-5.0; 2.0] | -3.4 [-6.7; 0.0] | 1.9 [-2.9; 6.7] | 120 | 121 |
| Dyspnea | 8.3 [4.8; 11.8] | 7.1 [3.8; 10.5] | 1.2 [-3.6; 6.0] | 119 | 121 |
| Insomnia | -8.7 [-11.9; -5.4] | -7.7 [-10.8; -4.5] | -1.0 [-5.5; 3.4] | 120 | 121 |
| Constipation | -1.9 [-5.2; 1.3] | -4.9 [-8.0; -1.7] | 2.9 [-1.6; 7.4] | 120 | 121 |
| Diarrhea | 4.1 [0.3; 8.0] | 1.2 [-2.5; 4.9] | 2.9 [-2.4; 8.2] | 119 | 120 |
| Financial difficulties | -3.4 [-5.8; -1.0] | -0.3 [-2.6; 2.1] | -3.1 [-6.5; 0.2] | 119 | 120 |
| QLQ-PAN26 ^e^ |  |  |  |  |  |
| Pancreatic pain | -4.0 [-7.8; -0.3] | -2.9 [-6.7; 0.9] | -1.1 [-4.7; 2.4] | 119 | 119 |
| Digestive symptoms | -1.1 [-4.7; 2.5] | -5.0 [-8.5; -1.5] | 3.9 [-1.1; 8.9] | 119 | 119 |
| Hepatic | 0.9 [-0.7; 2.4] | -1.2 [-2.6; 0.3] | 2.0 [-0.1; 4.1] | 119 | 119 |
| Altered bowel habit | 3.6 [0.2; 6.9] | 2.0 [-1.2; 5.2] | 1.5 [-3.1; 6.1] | 118 | 119 |
| Body image | 7.9 [4.0; 11.8] | 8.4 [4.7; 12.2] | -0.5 [-5.9; 4.8] | 119 | 120 |
| Satisfaction with health care | 3.2 [0.0; 6.5] | 1.5 [-1.6; 4.6] | 1.7 [-2.7; 6.2] | 118 | 120 |
| Sexuality | 4.7 [-0.2; 9.6] | 4.8 [0.0; 9.6] | -0.1 [-6.9; 6.7] | 115 | 112 |
| Bloated feeling in your abdomen | 0.9 [-3.0; 4.9] | 0.2 [-3.6; 4.0] | 0.7 [-4.7; 6.2] | 119 | 119 |
| Food and drink taste different | 7.5 [3.3; 11.7] | 5.8 [1.8; 9.9] | 1.7 [-4.1; 7.5] | 119 | 119 |
| Indigestion | -0.7 [-4.2; 2.9] | -3.9 [-7.3; -0.5] | 3.3 [-1.6; 8.2] | 117 | 118 |
| Bothered by gas (flatulence) | -4.1 [-8.1; -0.1] | -0.8 [-4.7; 3.1] | -3.3 [-8.9; 2.2] | 119 | 118 |
| Weak in your arms and legs | 11.7 [7.5; 15.9] | 12.8 [8.7; 16.9] | -1.1 [-6.9; 4.7] | 119 | 119 |
| Dry mouth | 5.7 [1.6; 9.8] | 3.1 [-0.8; 7.1] | 2.6 [-3.0; 8.3] | 118 | 119 |
| Troubled with side-effects | 13.3 [9.3; 17.4] | 8.7 [4.8; 12.6] | 4.6 [-0.9; 10.2] | 112 | 112 |
| Worried about future health | -6.6 [-10.4; -2.8] | -8.2 [-11.8; -4.5] | 1.6 [-3.6; 6.8] | 119 | 119 |
| Limited in planning activities | 3.0 [-1.3; 7.4] | 1.2 [-3.1; 5.4] | 1.9 [-4.2; 7.9] | 118 | 115 |

^a^ Values are difference by visit in health-related quality of life score from baseline estimated by mixed model regression.

^b^ Treatment effect refers to the difference in mean change from baseline between the mistletoe extract and the placebo arms estimated by mixed model regression. No statistically significant interaction effects between treatment and visit were observed for any of the scales

^c^ Number of patients with at least one baseline value and one from follow up visit for the specific item. Total number of patients in the follow-up analysis set is 241: Mistletoe extract 220 and Placebo 221.

^d^ Higher is better

^e^ Lower is better

^f^ Significant difference to the disadvantage of mistletoe extract in bold (p=0.02)

1. Wode K, Kienle GS, Björ O, Fransson P, Sharp L, Elander NO, Bernhardson B-M, Johansson B, Ardnor CE, Scheibling U: **Mistletoe extract in patients with advanced pancreatic cancer: a double-blind, randomized, placebo-controlled trial (MISTRAL)**. *J Deutsches Ärzteblatt International* 2024, **121**(11):347.

## S-Figure 1: Mean changes in quality-of-life scores for symtom scales.

Changes in quality of life scorescompared to baseline scores (95% confidence interval), calculated using mixed model regression. Data was collected using the EORTC-PAN-26 questionnaire.

Blue mistletoe extract, red placebo.

Lower scale indicate better qulatiy of life.

P-value from test of the treatment effect by Satterthwaite's method is shown in the figure.

**
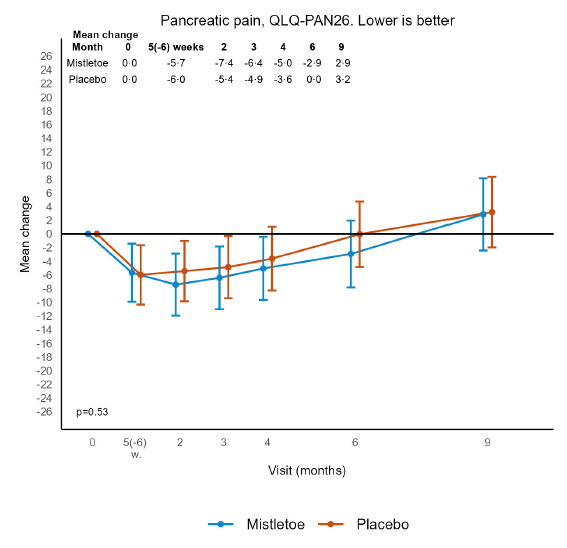
**

**
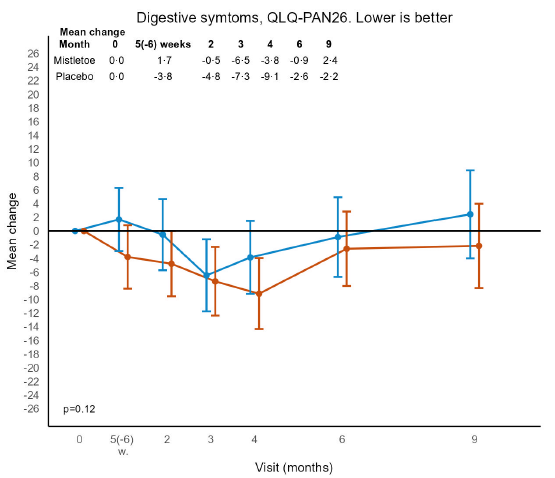
**

**
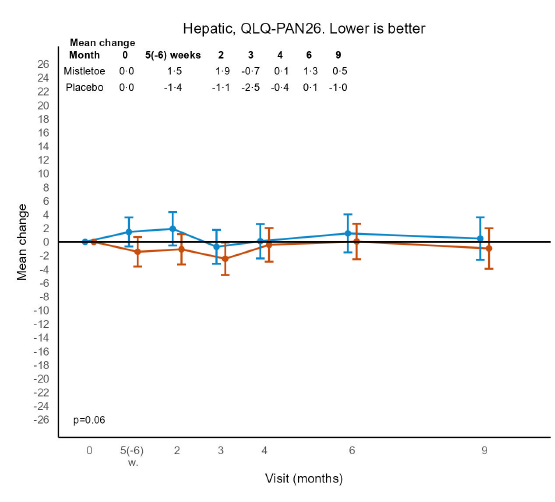
**

**
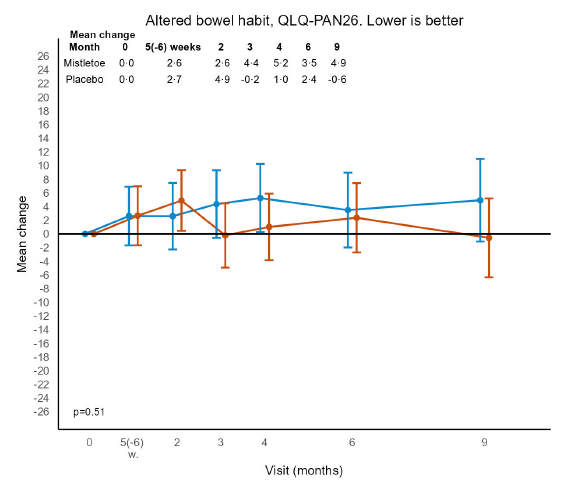
**

**
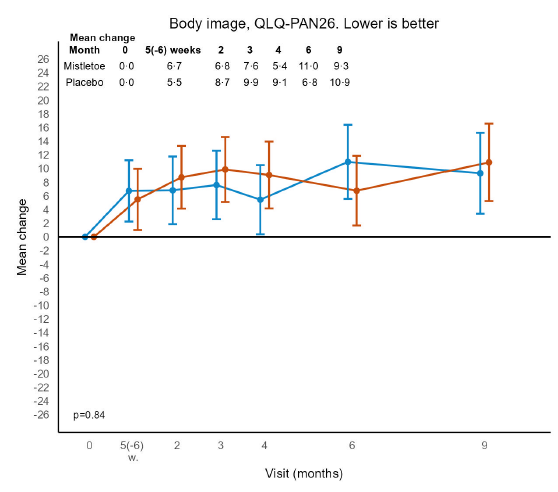
**

**
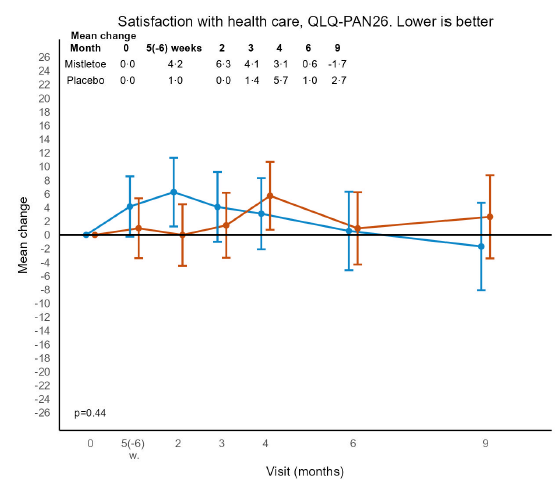
**

**
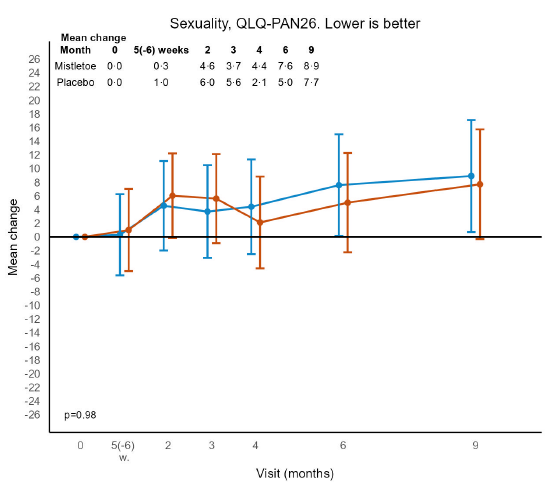
**

**
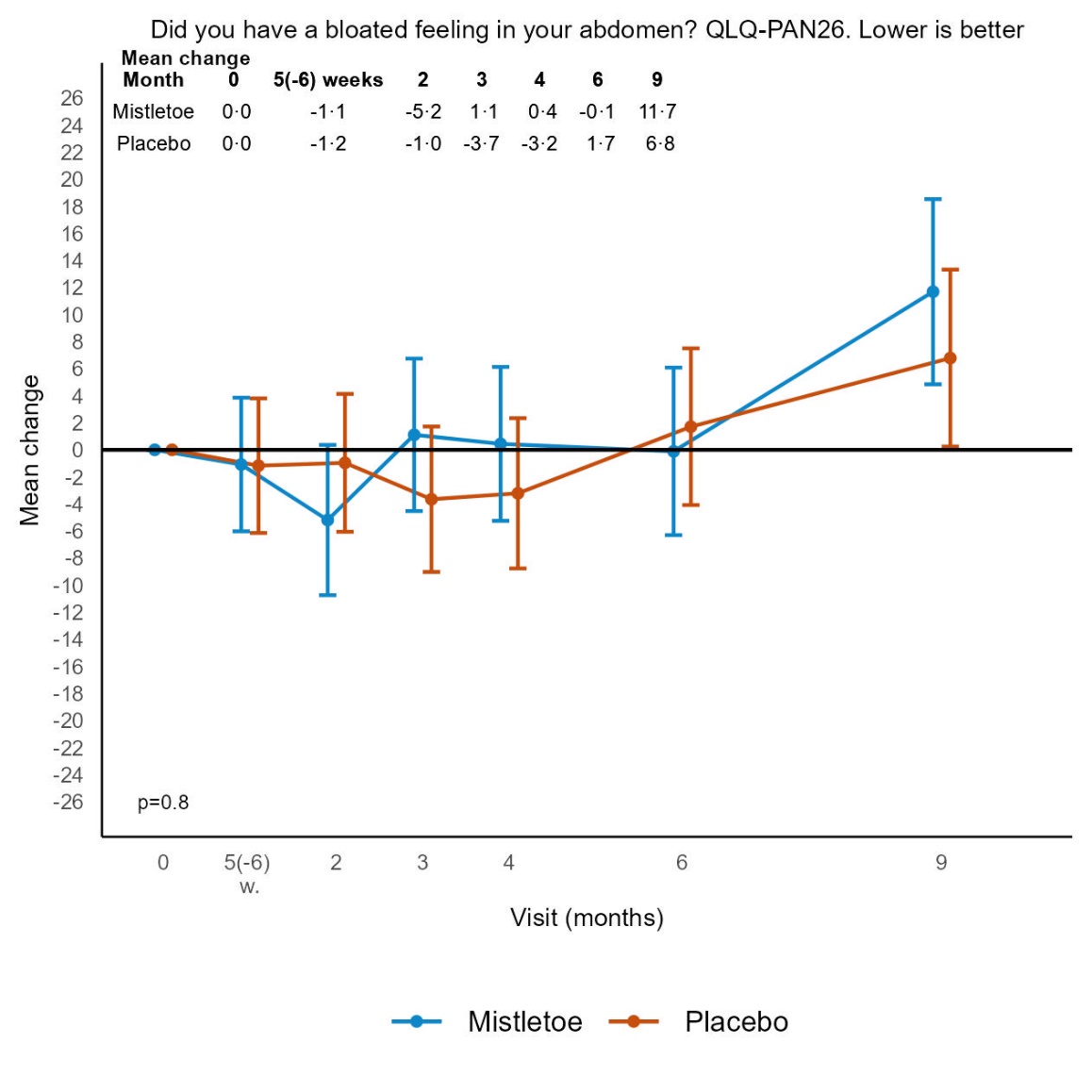
**

**
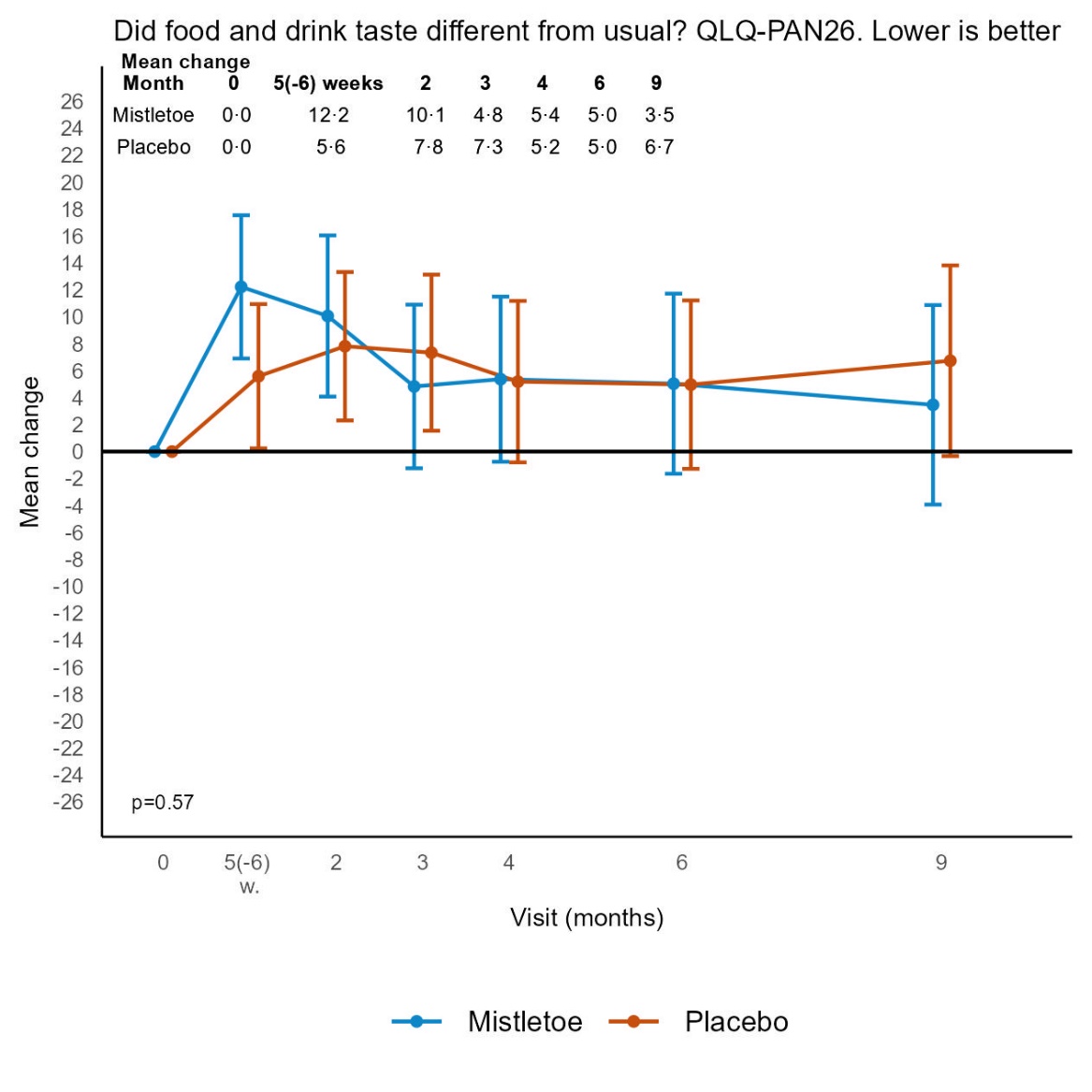
**

**
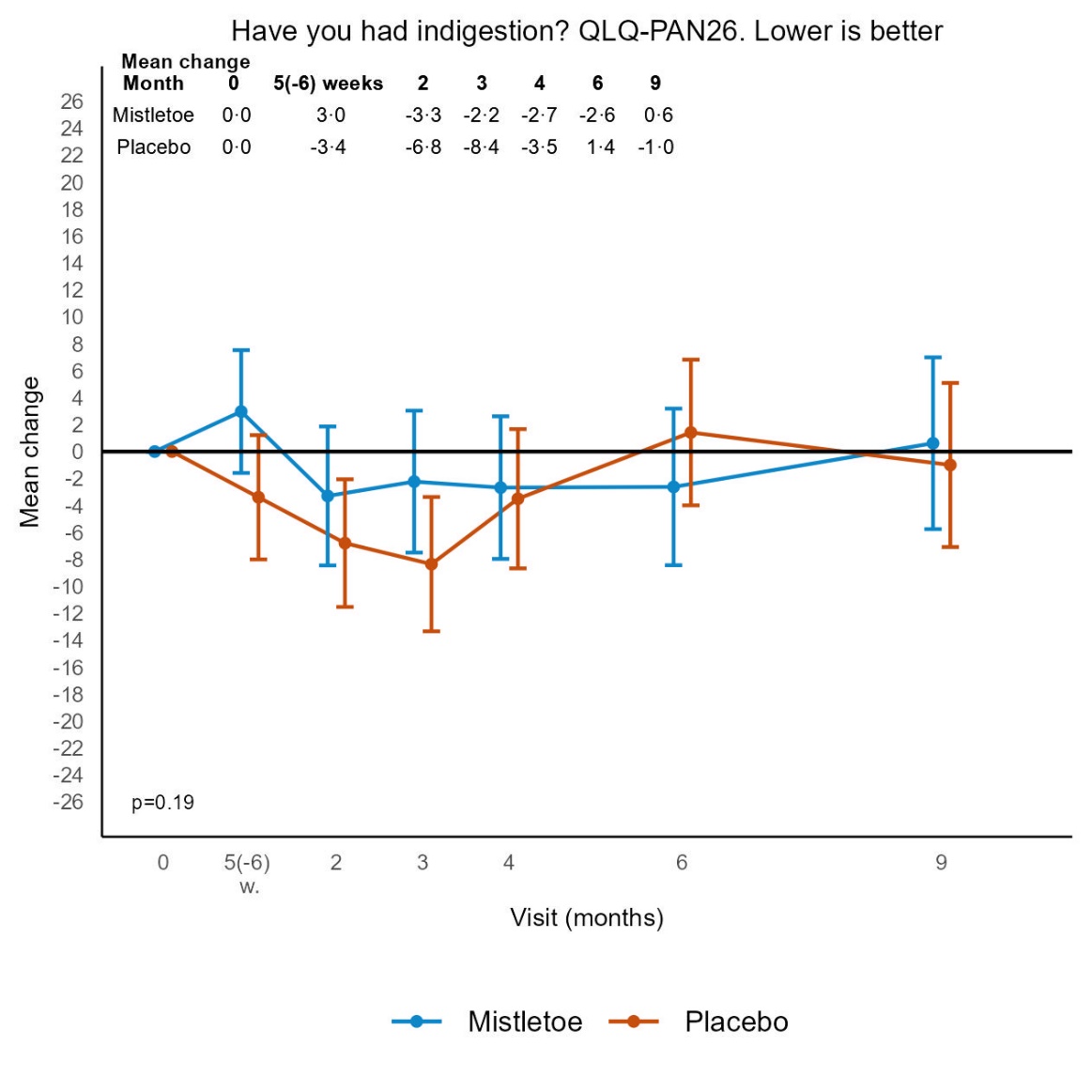
**

**
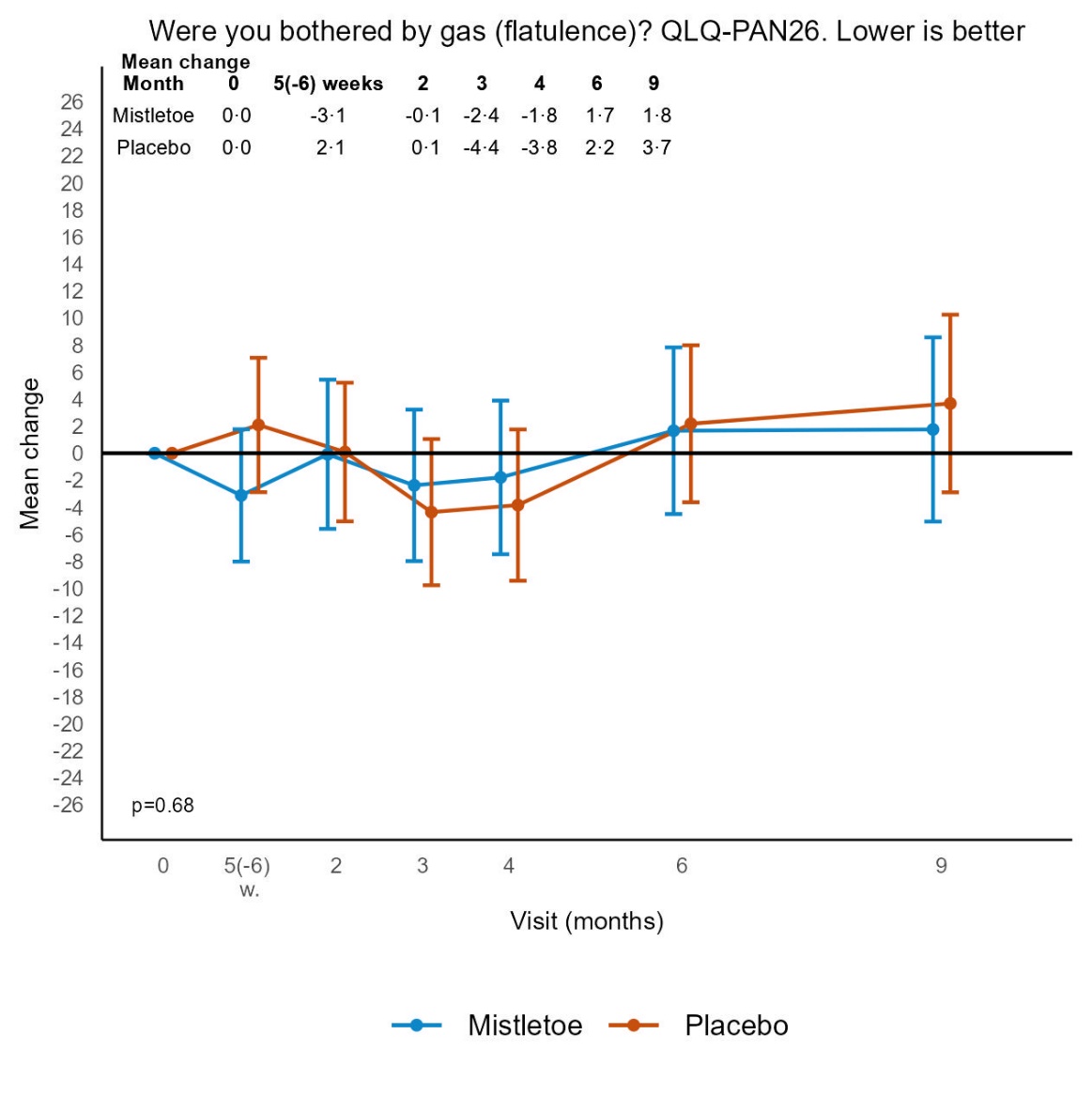
**

**
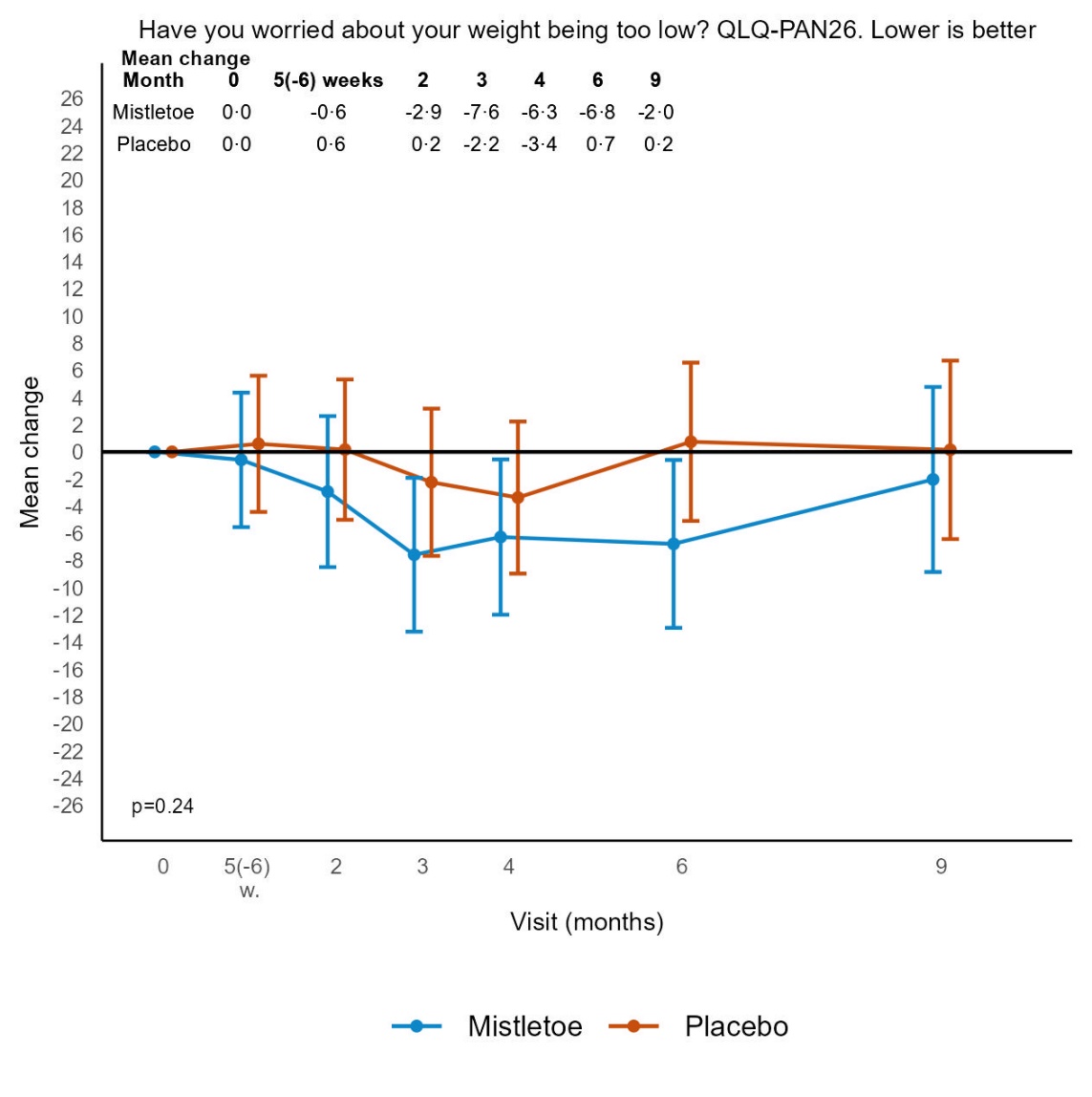
**

**
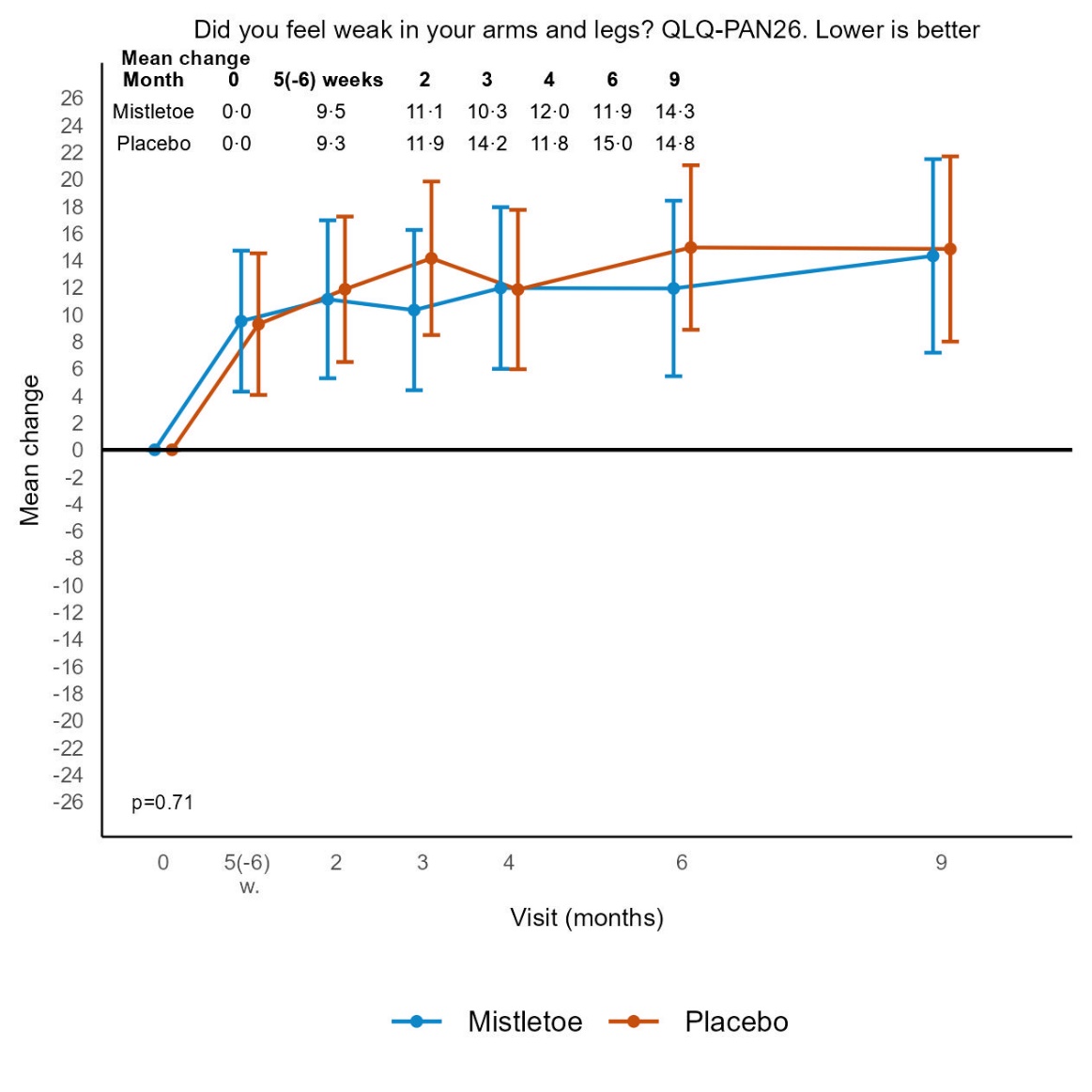
**

**
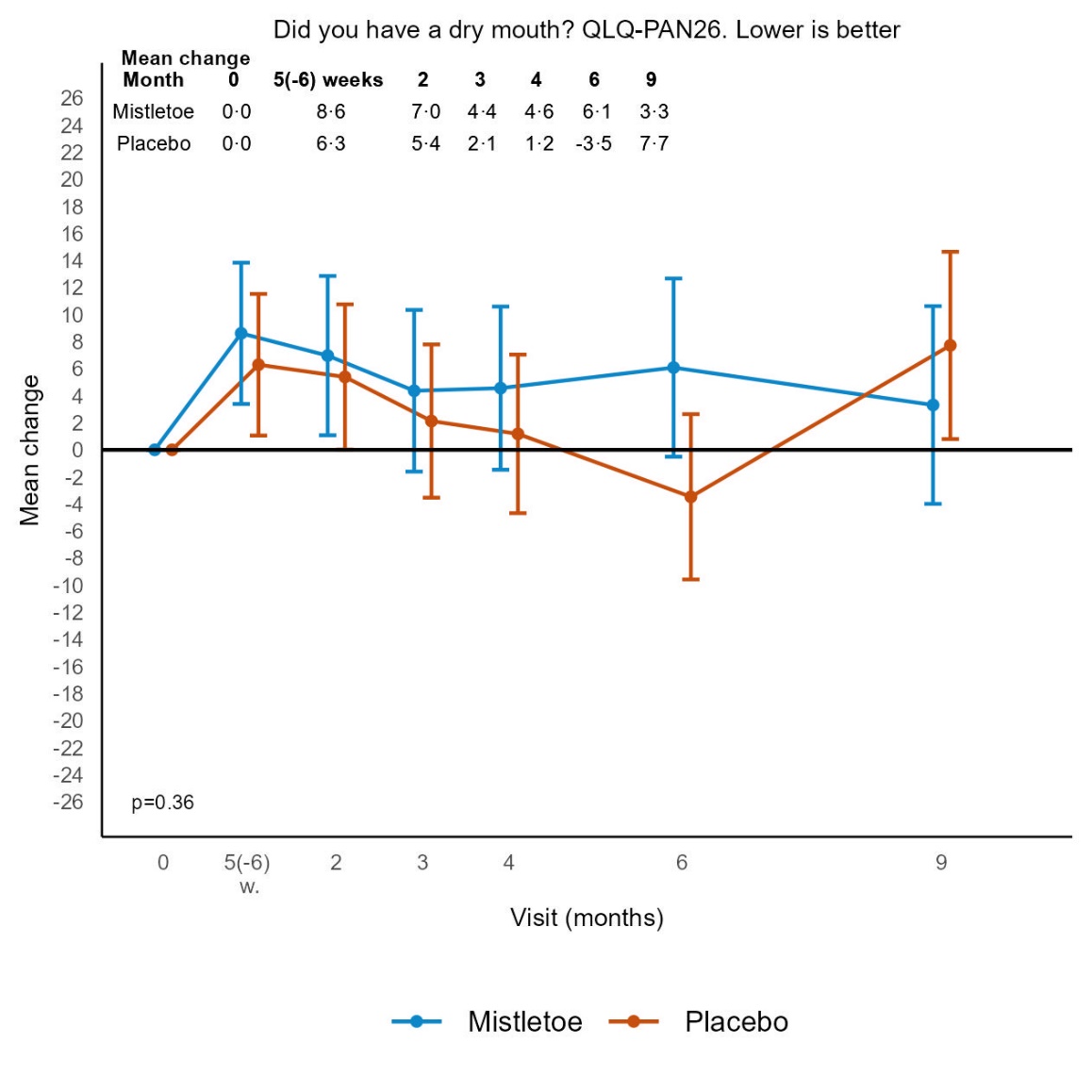
**

**
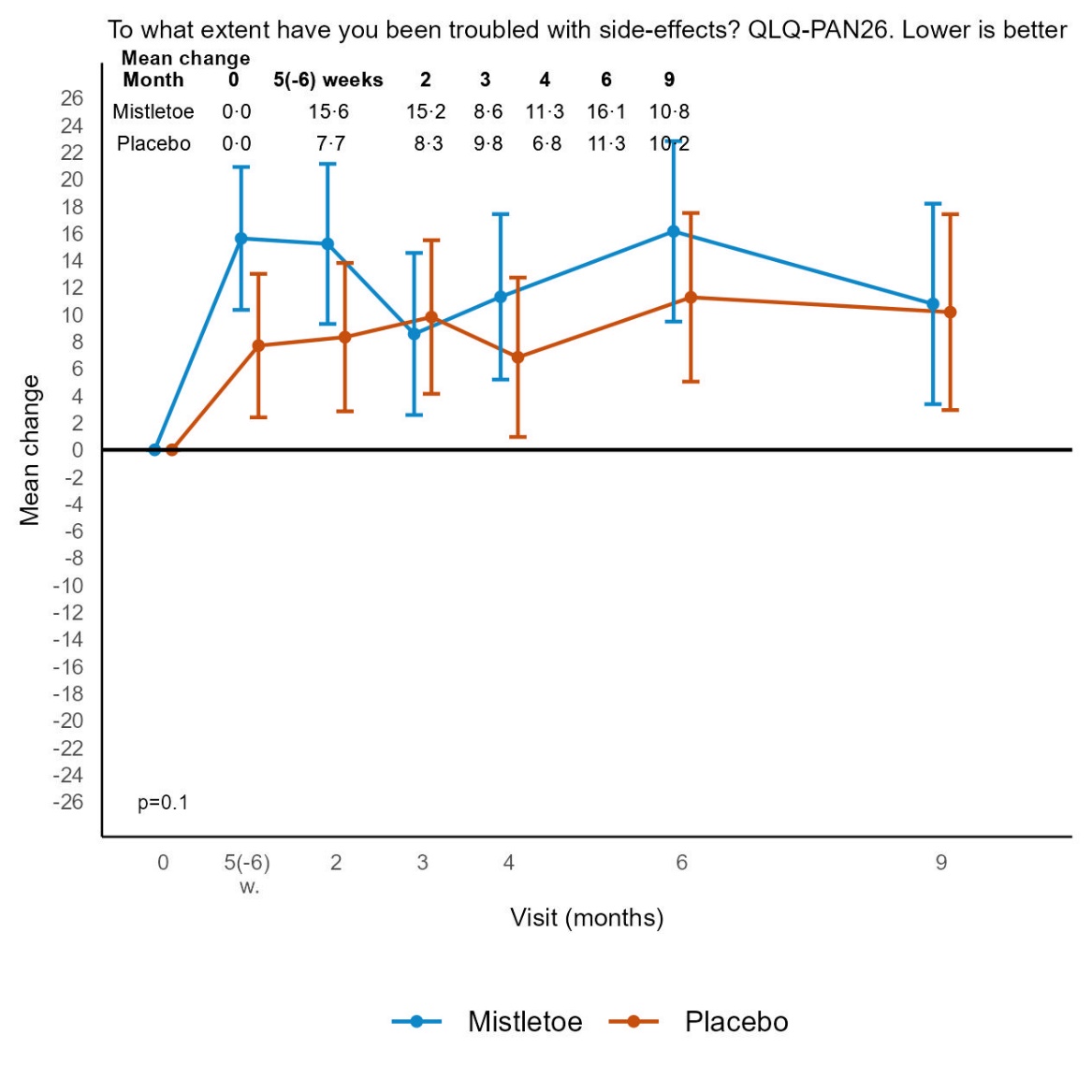
**

**
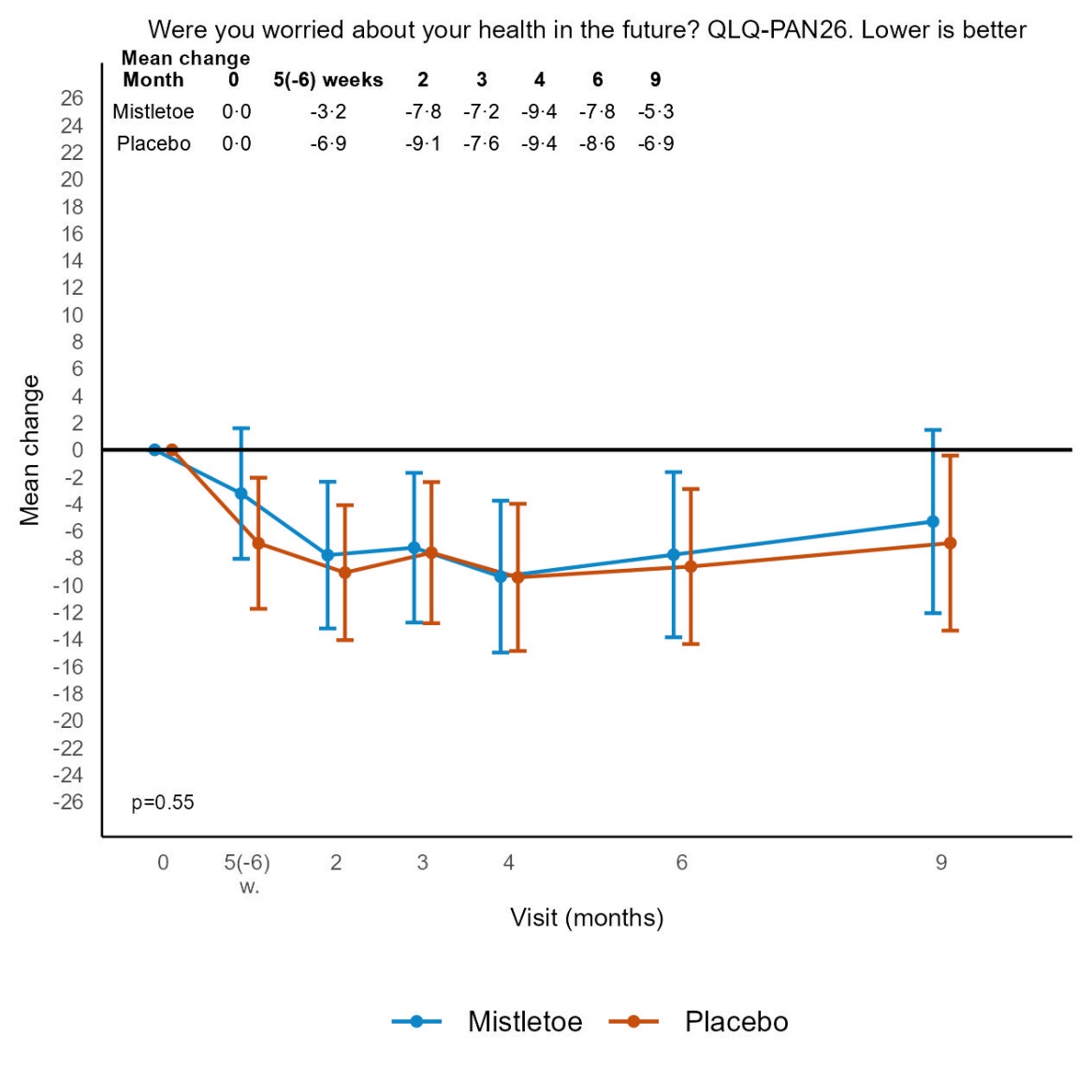
**

**
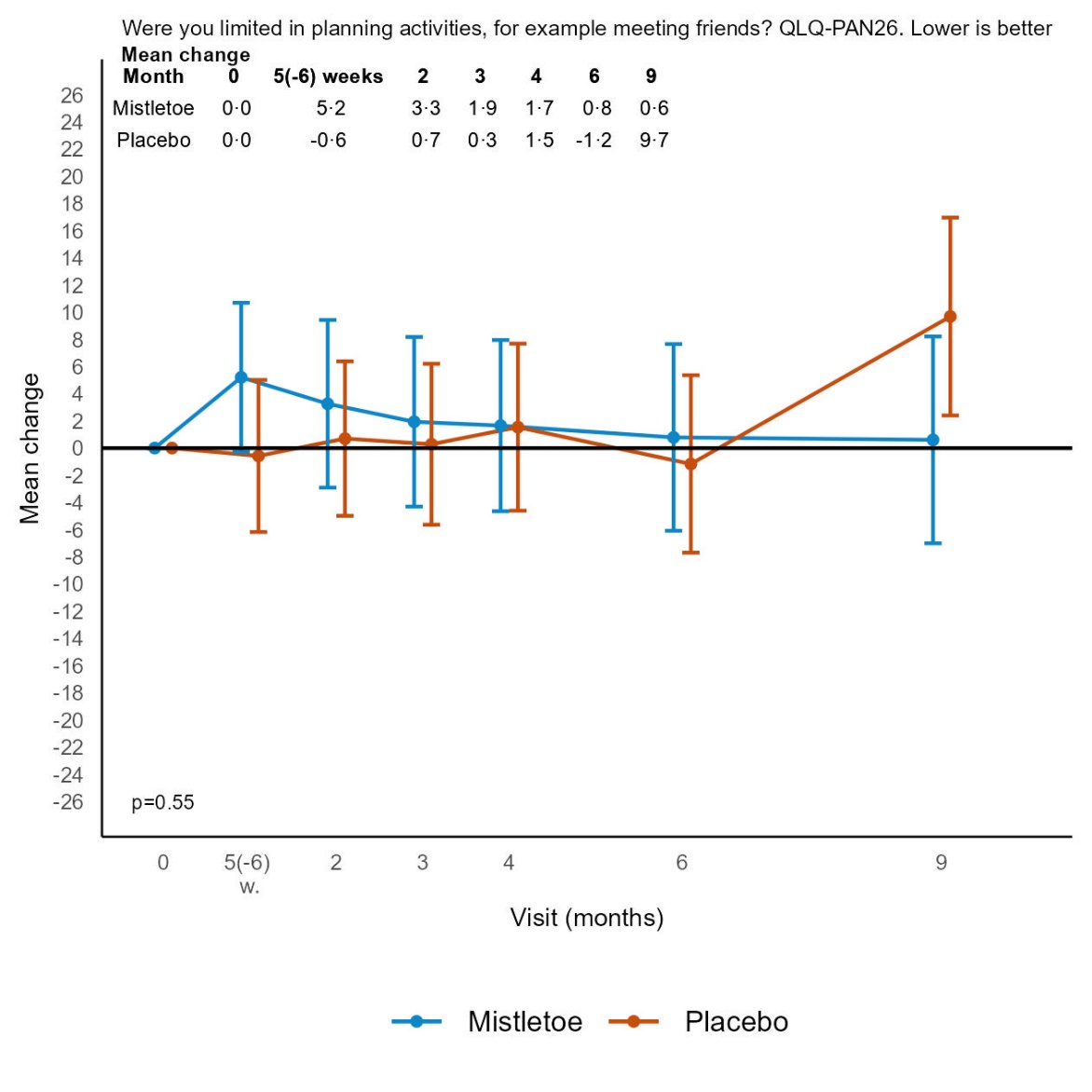
**

## S-Table 2: Test of difference

Test of difference between arbs in weight change per visit estimated from Mixed model regression. Overall test p=0.45. Interaction effect of treatment and time p=0.13

$contrasts

tfnf = 5(-6) weeks:

contrast estimate SE df t.ratio p.value

A - B -0.276 0.730 494 -0.379 0.7051

tfnf = 2:

contrast estimate SE df t.ratio p.value

A - B -0.591 0.773 563 -0.765 0.4446

tfnf = 3:

contrast estimate SE df t.ratio p.value

A - B -0.466 0.799 609 -0.583 0.5600

tfnf = 4:

contrast estimate SE df t.ratio p.value

A - B -0.243 0.823 657 -0.296 0.7675

tfnf = 6:

contrast estimate SE df t.ratio p.value

A - B -1.725 0.871 747 -1.980 0.0480

tfnf = 9:

contrast estimate SE df t.ratio p.value

A - B -2.432 0.972 903 -2.504 0.0125

## S-Table 3: Days in hospital during the study

Average number of days in hospital^1^ (including in-patient care at palliative wards) in absolute numbers and in relation to time (days) in study.

| **Treatment** | **Total number of days in study** | **Total number of days in hospital** | **Number of patients** | **Average number of days in hospital per patient** | **% Days in hospital of total number of days in study**^2^ |
| --- | --- | --- | --- | --- | --- |
| mistletoe extract | 126784 | 1145 | 143 | 8 | 0,9 |
| Placebo | 146471 | 1135 | 147 | 7,7 | 0,8 |
| Total | 273255 | 2280 | 290 | 7,9 | 0,8 |

^1^ Except planned stays such as for chemotherapy

^2^Time in study is defined as time from baseline visit to discontinued intervention or end of nine-months treatment period.

## S-Table 4: Days in hospital since previous visit

Average number of days in hospital since previous visit (number of patients) by treatment arm

| **Treatment** | **5(-6) weeks** | **Month2** | **Month3** | **Month4** | **Month6** | **Month9** | **Early Withdrawal** |
| --- | --- | --- | --- | --- | --- | --- | --- |
| mistletoe extract | 1.3 (126) | 0.85 (101) | 1.05 (90) | 1.75 (82) | 1.75 (71) | 0.8 (51) | 5.51 (92) |
| Placebo | 0.82 (125) | 0.92 (115) | 0.97 (107) | 1.02 (95) | 1.02 (82) | 1.43 (61) | 6.98 (86) |
| Total | 1.04 (251) | 0.89 (216) | 1.01 (197) | 1.36 (177) | 1.36 (153) | 1.14 (112) | 6.2 (178) |

## S-Table 5: Concomitant medication for symptom relief

Number and percentage of all patients^1^ with concomitant medication^2^ for symptom relief by indication

|  | **Number of patients** | | | **Percent** | |  |
| --- | --- | --- | --- | --- | --- | --- |
| **Indication** | **Mistletoe extract** | **Placebo** | **Total** | **Mistletoe extract** | **Placebo** | **Total** |
| Anxiety | 23 | 20 | 43 | 16,1 | 13,6 | 14,8 |
| Depression | 21 | 25 | 46 | 14,7 | 17,0 | 15,9 |
| Depression/neuropathic pain | 5 | 2 | 7 | 3,5 | 1,4 | 2,4 |
| Infection- bacteria | 54 | 59 | 113 | 37,8 | 40,1 | 39,0 |
| Infection – fungus | 42 | 50 | 92 | 29,4 | 34,0 | 31,7 |
| Infection - virus | 1 | 1 | 2 | 0,7 | 0,7 | 0,7 |
| Insomnia | 27 | 26 | 53 | 18,9 | 17,7 | 18,3 |
| Nausea/vomiting | 39 | 37 | 76 | 27,3 | 25,2 | 26,2 |
| Neuropathic pain | 21 | 11 | 32 | 14,7 | 7,5 | 11,0 |
| Pain-mild to moderate | 63 | 69 | 132 | 44,1 | 46,9 | 45,5 |
| Pain-moderate to severe | 98 | 103 | 201 | 68,5 | 70,1 | 69,3 |

^1^ Mistletoe extract: 143, Placebo: 147, Total: 290 ^2^ exclusive on-demand use/prescription. Data for end-of-life medication with continuous infusion and/or on-demand doses not shown.

## S-Table 6: Patient-initiated use of natural products, vitamins and minerals^1^

Number and percentage of all patients (mistletoe extract n=143, placebo n=147, total n=290) who reported use on at least one occasion.

|  | **mistletoe extract** | **Placebo** | **Total** |
| --- | --- | --- | --- |
| **Number of patients** | 26 | 31 | 57 |
| **Percent** | 18,2 | 21,1 | 19,7 |

^1^ vitamins and minerals prescribed by a doctor are not included

## S-Table 7: Glucocorticoid use for symptom management^1^.

Shown in months per treatment arm.

| **Treatment arm** | **Min** | **1st Qu.** | **Median** | **Mean** | **3rd Qu.** | **Max** | **Number of patients** |
| --- | --- | --- | --- | --- | --- | --- | --- |
| Mistletoe extract | 0.03 | 0.43 | 1.15 | 1.91 | 2.17 | 8.94 | 69 |
| Placebo | 0.03 | 0.43 | 1.12 | 1.83 | 2.66 | 6.94 | 61 |
| Total | 0.03 | 0.43 | 1.12 | 1.87 | 2.52 | 8.94 | 130 |

^1^E.g. nausea, appetite loss, pain, improvement of general condition, cholestasis.

## S-Figure 2: Glucocorticoid use by time in study per indication

Percent glucocorticoid use by time in study shown per indication (number of patients treated for the specific symptom). For each box, time was calculated for patients per indication. Examples for “Other” are increased liver enzymes, brain metastases, tumour fever. Time in study was defined as from randomization to earliest of time to death, discontinued intervention or nine months of follow-up.

## S-Figure 3 A-B: Eosinophil counts and albumin levels

Comparison of eosinophil counts (A) and albumin levels (B) in participants receiving either placebo or mistletoe extract at baseline visit before the first injection with placebo (left) or mistletoe extract mistletoe extract (right) and month one, two, three to five and nine to ten. Median 95% CI is given. Significant difference between placebo and mistletoe extract arm at two months (# p < 0.05), three to five months (## p < 0.01) and nine to ten months (### p < 0.001)

## S-Figure 4 A-D: Eosinophil- and lymphocyte counts, Ca19-9 and albumin levels

Comparison of eosinophil (A) and lymphocyte counts (B), Ca19-9 (C), and albumin levels (D) in participants receiving placebo (upper diagrams) or mistletoe extract (lower diagrams) at baseline visit and month one, two, three to five and nine to ten in relation to best supportive care (left diagrams) or chemotherapy (right diagrams). Median 95% CI is given. Significant as compared to the mistletoe extract arm at the same measurement: # p < 0.05, ## p < 0.01, ### p < 0.001. Significant as compared to participants with best supportive care at the same measurement: * p < 0.05

## S-Table 8: Comparison of laboratory parameters in mistletoe extract versus placebo arm, unpaired analysis

Comparison of p-values for laboratory parameters in participants receiving either mistletoe extract or placebo (regardless treatment with chemotherapy or best supportive therapy) at baseline visit, month one, two, three to five and nine to ten. P< 0.05 was considered statistically significant (bold grey)

| **Mistletoe extract versus placebo** | | | | | |
| --- | --- | --- | --- | --- | --- |
| Laboratory parameters | Baseline | Month 1 | Month 2 | Month 3-5 | Month 9-10 |
|  | p-values | | | | |
| Leukocytes | 0,401 | 0,938 | 0,657 | 0,742 | 0,874 |
| Neutrophils | 0,400 | 0,604 | 0,318 | 0,308 | 0,975 |
| Eosinophils | 0,147 | 0,491 | **0,002** | **0,001** | **0,022** |
| Basophils | 0,387 | 0,976 | **0,045** | 0,086 | 0,693 |
| Lymphocytes | 0,731 | 0,144 | 0,082 | 0,239 | 0,302 |
| Monocytes | 0,436 | 0,727 | 0,860 | 0,701 | 0,811 |
| CRP | 0,818 | 0,761 | 0,741 | 0,112 | 0,655 |
| Albumin | 0,250 | 0,947 | 0,649 | 0,905 | 0,233 |
| Ca-19-9 | 0,819 | 0,267 | 0,139 | 0,365 | 0,882 |
| CD3 +. T-cells (%) | 0,378 | 0,520 | 0,979 | 0,286 | 0,298 |
| CD3 + T-cells (count) | 0,705 | 0,215 | 0,260 | 0,956 | 0,586 |
| CD19 + B-cells (%) | 0,659 | 0,369 | 0,756 | 0,158 | 0,869 |
| CD19 + B-Cells (count) | 0,558 | 0,126 | 0,588 | 0,250 | 0,609 |
| CD16+/CD56+ NK-cells (%) | 0,996 | **0,035** | 0,477 | 0,909 | 0,102 |
| CD16+/CD56+ NK-cells (count) | 0,428 | 0,561 | 0,558 | 0,572 | 0,248 |
| CD4+/CD3+ T-cells (%) | 0,726 | 0,358 | 0,790 | 0,490 | 0,089 |
| CD4+/CD3+ T-cells (count) | 0,389 | 0,878 | 0,544 | 0,841 | 0,597 |
| CD8+/CD3+ T-cells (%) | 0,609 | 0,346 | 0,271 | 0,438 | 0,509 |
| CD8+/CD3+ T-cells (count) | 0,586 | 0,525 | 0,202 | 0,653 | 0,418 |
| CD4/CD8 Ratio | 0,493 | 0,287 | 0,741 | 0,534 | 0,298 |

## S-Table 9: Comparison of laboratory parameters in mistletoe extract versus placebo arm, paired analysis.

Comparison of p-values for laboratory parameters in participants receiving either mistletoe extract or placebo (regardless treatment with chemotherapy or best supportive care) at baseline visit (before first injection with study drug) versus month one, two, three to five and nine to ten. P< 0.05 was considered statistically significant (bold grey). For parameters shown in both count and percent, both had to have p<0.05 to be considered significant

| Laboratory parameters | **Placebo** | | | | | **Mistletoe extract** | | | |
| --- | --- | --- | --- | --- | --- | --- | --- | --- | --- |
|  | **Baseline versus** | | | | | | | | |
|  | Month 1 | Month 2 | Month 3-5 | Month 9-10 | Month 1 | | Month 2 | Month 3-5 | Month 9-10 |
|  | p-values | | | | | | | | |
| Leukocytes | 0,567 | 0,712 | 0,200 | 0,255 | 0,289 | | 0,661 | 0,263 | 0,831 |
| Neutrophils | 0,891 | 0,925 | 0,218 | 0,052 | 0,392 | | 0,931 | 0,477 | 0,722 |
| Eosinophils | 0,710 | 0,293 | 0,707 | **0,023** | 0,480 | | 0,091 | **0,012** | 0,077 |
| Basophils | 0,334 | 0,496 | 0,066 | 0,180 | 1,000 | | 0,276 | 0,317 | 0,317 |
| Lymphocytes | 0,179 | 0,421 | 0,314 | 0,484 | 0,298 | | 0,762 | 0,567 | 0,078 |
| Monocytes | 0,961 | 0,199 | 0,873 | 0,932 | 0,110 | | 0,154 | **0,027** | 0,462 |
| CRP | 0,405 | 0,148 | 0,139 | 0,345 | 0,317 | | 0,643 | 0,751 | 0,535 |
| Albumin | **0,05** | **0,004** | **0,019** | 0,234 | **0,000** | | **0,003** | **0,005** | **0,004** |
| Ca-19-9 | 0,342 | 0,125 | 0,471 | 0,554 | 0,150 | | 0,356 | 0,922 | 0,570 |
| CD3 + T-cells (%) | 0,330 | 0,918 | 0,287 | 0,690 | 0,353 | | 0,878 | 0,861 | 0,660 |
| CD3 + T-cells (count) | 0,186 | 0,830 | 0,692 | 0,469 | 0,245 | | 0,778 | 0,399 | 0,795 |
| CD19 + B-cells (%) | 0,220 | 0,897 | 0,296 | 0,278 | 0,714 | | 0,497 | 0,744 | **0,014** |
| CD19 + B-Cells (count) | **0,005** | 0,539 | **0,025** | 0,279 | 0,423 | | 0,431 | 0,861 | 0,522 |
| CD16+/CD56+ NK-cells (%) | 0,041 | 0,982 | 0,753 | 0,704 | 0,404 | | 0,984 | 0,777 | 0,114 |
| CD16+/CD56+ NK-cells (count) | 0,731 | 0,906 | 0,538 | 0,955 | 0,128 | | 0,708 | 0,820 | 0,422 |
| CD4+/CD3+ T-cells (%) | 0,189 | 0,747 | 0,588 | 0,682 | 0,761 | | 0,652 | 0,968 | 0,141 |
| CD4+/CD3+ T-cells (count) | 0,051 | 0,708 | 0,569 | 0,460 | 0,158 | | 0,776 | 0,316 | 0,067 |
| CD8+/CD3+ T-cells (%) | 0,774 | 0,303 | 0,749 | 0,529 | 0,384 | | 0,697 | 0,812 | 0,527 |
| CD8+/CD3+ T-cells (count) | 0,497 | 0,906 | 0,750 | 0,301 | 0,497 | | 0,670 | 0,259 | 0,255 |
| CD4/CD8 Ratio | 0,091 | 0,292 | 0,864 | 0,776 | 0,480 | | 0,845 | 0,970 | 0,064 |

## S-Table 10: Comparison of laboratory parameters in relation to treatment with best supportive care versus chemotherapy regardless treatment arm, unpaired analysis.

Comparison of p-values for laboratory parameters in patients receiving best supportive care or palliative chemotherapy at baseline visit, month one, two, three to five and nine to ten. P< 0.05 considered as statistically significant (grey bolt)

| **Laboratory parameters** | Best supportive care | | | | | Chemotherapy | | | | |
| --- | --- | --- | --- | --- | --- | --- | --- | --- | --- | --- |
|  | Baseline | Month 1 | Month 2 | Month 3-5 | Month 9-10 | Baseline | Month 1 | Month 2 | Month 3-5 | Month 9-10 |
|  | p-values | | | | | | | | | |
| Leukocytes | 0,186 | 0,462 | 0,578 | 0,769 | 0,241 | 0,938 | 0,734 | 0,687 | 0,880 | 0,475 |
| Neutrophils | 0,561 | 0,664 | 0,642 | 0,420 | 1,0000 | 0,598 | 0,282 | 0,250 | 0,734 | 0,765 |
| Eosinophils | 0,879 | 0,298 | 0,059 | **0,007** | 0,731 | 0,112 | 0,705 | **0,010** | **0,005** | **0,008** |
| Basophils | 1,000 | 1,000 | 1,000 | 1,000 | 0,564 | 0,308 | 0,893 | **0,034** | **0,043** | 0,850 |
| Lymphocytes | 0,283 | 0,347 | 0,518 | 0,769 | 0,505 | 0,224 | **0,009** | **0,015** | **0,072** | 0,963 |
| Monocytes | 0,064 | 0,564 | 0,573 | 0,497 | 0,280 | 0,688 | 0,466 | 1,000 | 0,669 | 0,729 |
| CRP | 0,815 | 0,546 | 0,774 | 0,497 | 0,252 | 0,851 | 0,763 | 0,489 | **0,008** | 0,678 |
| Albumin | 0,484 | 0,736 | 0,352 | 0,457 | 0,731 | 0,295 | 0,813 | 0,868 | 0,850 | 0,445 |
| Ca-19-9 | 0,501 | 0,570 | 0,814 | 0,308 | 0,617 | 0,528 | 0,294 | 0,096 | 0,318 | 0,645 |
| CD3 + T-cells (%) | 0,218 | 0,441 | 0,780 | 0,339 | 0,615 | 0,743 | 0,289 | 0,929 | 0,529 | 0,231 |
| CD3 + T-cells (count) | 0,057 | 0,151 | 0,405 | 0,306 | 0,182 | 0,094 | **0,022** | 0,067 | 0,631 | 0,542 |
| CD19 + B-cells (%) | 0,559 | 0,763 | 0,307 | 0,659 | **0,044** | 0,994 | 0,539 | 0,819 | 0,226 | 0,304 |
| CD19 + B-Cells (count) | 0,364 | 0,615 | 0,926 | 0,941 | 0,317 | 0,288 | **0,047** | 0,770 | 0,204 | 0,525 |
| CD16+/CD56+ NK-cells (%) | 0,470 | 0,894 | 0,926 | 0,769 | 0,736 | 0,792 | **0,013** | 0,683 | 0,573 | 0,231 |
| CD16+/CD56+ NK-cells (count) | 0,944 | 0,973 | 0,926 | 0,660 | 0,399 | 0,285 | 0,648 | 0,257 | 0,677 | 0,678 |
| CD4+/CD3+ T-cells (%) | 0,942 | 0,366 | 0,459 | 0,607 | 0,402 | 0,609 | 0,119 | 0,979 | 0,611 | 0,143 |
| CD4+/CD3+ T-cells (count) | 0,088 | **0,038** | 0,115 | 0,305 | 0,317 | 0,072 | **0,022** | 0,559 | 0,886 | 0,733 |
| CD8+/CD3+ T-cells (%) | 0,478 | 0,815 | 0,643 | 1,000 | 0,317 | 0,483 | 0,885 | 0,979 | 0,604 | 0,558 |
| CD8+/CD3+ T-cells (count) | 0,083 | 0,525 | 0,644 | 0,714 | 0,182 | 0,579 | 0,173 | 0,228 | 0,326 | 0,961 |
| CD4/CD8 Ratio | 0,591 | 0,616 | 0,518 | 0,770 | 0,182 | 0,483 | 0,240 | 0,812 | 0,559 | 0,367 |

## S-Table 11: Comparison of p-values for laboratory parameters for best supportive care versus chemotherapy in mistletoe extract and placebo arm, paired analysis

Comparison of laboratory parameters at baseline visit before start with first injection with study drug and month one, two, three to five and nine to ten in relation to treatment with best supportive care or chemotherapy. P< 0.05 was considered statistically significant (grey bolt)

| Laboratory parameters | **Placebo** | | | | **Mistletoe extract** | | | |
| --- | --- | --- | --- | --- | --- | --- | --- | --- |
|  | **Baseline versus** | | | | | | | |
|  | Month 1 | Month 2 | Month 3–5 | Month 9–10 | Month 1 | Month 2 | Month 3–5 | Month 9–10 |
|  | p-values | | | | | | | |
| **Best supportive care** | | | | | | | | |
| Leukocytes | 0,508 | 0,172 | **0,043** | 0,461 | 0,051 | 0,715 | 0,225 | 0,655 |
| Neutrophils | 0,476 | **0,033** | 0,074 | 0,104 | 0,093 | 0,715 | 0,588 | 0,655 |
| Eosinophils | 0,739 | 0,705 | 0,655 | **0,046** | 0,084 | 0,102 | **0,043** | 0,180 |
| Basophils | 1,000 | 1,000 | 1,000 | 0,317 | 1,000 | 1,000 | 1,000 | 1,000 |
| Lymphocytes | 0,838 | 0,673 | 0,248 | 0,225 | 0,944 | 0,180 | 0,194 | 0,317 |
| Monocytes | 0,201 | 0,888 | 0,104 | 0,414 | 0,566 | 0,180 | 0,180 | 0,317 |
| CRP | 0,779 | 0,273 | 0,655 | 0,180 | 0,327 | 0,180 | 0,109 | 0,317 |
| Albumin | 0,797 | 0,574 | 0,750 | 0,705 | 0,237 | 1,000 | 0,336 | 0,317 |
| Ca-19-9 | **0,047** | 0,207 | 0,345 | **0,042** | **0,017** | 0,285 | 0,068 | 0,180 |
| CD3 + T-cells (%) | 0,156 | 0,932 | 0,684 | 0,892 | 0,260 | 0,180 | 0,066 | 0,655 |
| CD3 + T-cells (count) | 0,563 | 0,635 | 0,600 | 0,138 | 0,594 | 0,109 | 0,686 | 0,655 |
| CD19 + B-cells (%) | 0,931 | 1,000 | 0,317 | 0,705 | 0,202 | 1,000 | 0,197 | 0,655 |
| CD19 + B-Cells (count) | 0,091 | 0,865 | **0,042** | 0,680 | 0,271 | 0,141 | 0,102 | 0,180 |
| CD16+/CD56+ NK-cells (%) | 0,152 | 0,906 | 0,917 | 0,891 | **0,034** | 0,197 | 0,141 | 0,180 |
| CD16+/CD56+ NK-cells (count) | 0,959 | 0,906 | 0,752 | 0,588 | 0,206 | 0,066 | 0,104 | 0,180 |
| CD4+/CD3+ T-cells (%) | 0,154 | 0,766 | 1,000 | 0,492 | **0,011** | 0,414 | 0,581 | 0,180 |
| CD4+/CD3+ T-cells (count) | 0,091 | 0,484 | 0,600 | 0,138 | 0,213 | 0,109 | 0,285 | 0,655 |
| CD8+/CD3+ T-cells (%) | 0,058 | 0,168 | 0,102 | 0,715 | **0,017** | 0,180 | 0,066 | 0,317 |
| CD8+/CD3+ T-cells (count) | 1,000 | 0,833 | 0,596 | 0,223 | 0,141 | 0,285 | 1,000 | 0,317 |
| CD4/CD8 Ratio | **0,041** | 0,767 | 0,600 | 0,893 | 0,086 | 0,066 | 0,225 | 0,180 |
| **Chemotherapy** | | | | | | | | |
| Leukocytes | 0,855 | 0,431 | 0,476 | 0,158 | 0,851 | 0,777 | 0,486 | 0,755 |
| Neutrophils | 0,915 | 0,484 | 0,411 | 0,133 | 0,939 | 0,981 | 0,543 | 0,865 |
| Eosinophils | 0,819 | 0,341 | 0,507 | 0,168 | 0,088 | 0,393 | 0,068 | 0,135 |
| Basophils | 0,334 | 0,496 | 0,066 | 0,317 | 1,000 | 0,276 | 0,317 | 0,317 |
| Lymphocytes | 0,130 | 0,352 | 0,639 | 0,688 | 0,296 | 0,943 | 0,321 | **0,038** |
| Monocytes | 0,594 | 0,193 | 0,455 | 0,694 | 0,119 | 0,221 | 0,052 | 0,504 |
| CRP | 0,436 | 0,242 | 0,091 | 0,504 | 0,429 | 0,962 | 0,758 | 0,513 |
| albumin | **0,038** | **0,002** | **0,006** | 0,123 | **0,000** | **0,001** | **0,001** | **0,005** |
| Ca-19-9 | 0,986 | **0,024** | 0,259 | 0,754 | 0,615 | 0,158 | 0,384 | 0,701 |
| CD3 + T-cells (%) | 0,493 | 0,927 | 0,270 | 0,759 | 0,144 | 0,640 | 0,285 | 0,730 |
| CD3 + T-cells (count) | 0,223 | 0,710 | 0,498 | 0,722 | 0,269 | 0,469 | 0,218 | 0,865 |
| CD19 + B-cells (%) | 0,154 | 0,844 | 0,348 | 0,349 | 0,968 | 0,430 | 0,774 | **0,017** |
| CD19 + B-Cells (count) | **0,023** | 0,376 | 0,161 | 0,284 | 0,696 | 0,256 | 0,378 | 0,477 |
| CD16+/CD56+ NK-cells (%) | 0,126 | 0,968 | 0,616 | 0,634 | **0,044** | 0,377 | 0,668 | 0,197 |
| CD16+/CD56+ NK-cells (count) | 0,680 | 0,986 | 0,445 | 0,414 | **0,007** | 0,154 | 0,275 | 0,572 |
| CD4+/CD3+ T-cells (%) | 0,526 | 0,830 | 0,520 | 1,000 | 0,290 | 0,900 | 0,842 | 0,350 |
| CD4+/CD3+ T-cells (count) | 0,217 | 0,577 | 0,338 | 0,799 | 0,383 | 0,402 | 0,118 | 0,060 |
| CD8+/CD3+ T-cells (%) | 0,452 | 0,594 | 0,985 | 0,646 | 0,656 | 0,431 | 0,379 | 0,455 |
| CD8+/CD3+ T-cells (count) | 0,389 | 0,927 | 0,592 | 0,789 | 0,153 | 0,975 | 0,198 | 0,168 |
| CD4/CD8 Ratio | 0,465 | 0,265 | 0,897 | 0,625 | 0,853 | 0,594 | 0,629 | 0,136 |
